# Male and female contributions to behavioral isolation in darters as a function of genetic distance and color distance

**DOI:** 10.1111/evo.13321

**Published:** 2017-09-14

**Authors:** Rachel L. Moran, Muchu Zhou, Julian M. Catchen, Rebecca C. Fuller

**Affiliations:** ^1^ Department of Animal Biology University of Illinois Champaign Illinois 61820; ^2^ Program in Ecology, Evolution, and Conservation Biology University of Illinois Champaign Illinois 61820

**Keywords:** Behavioral isolation, color pattern, genetic distance, population divergence, reinforcement, speciation, sexual selection

## Abstract

Determining which reproductive isolating barriers arise first between geographically isolated lineages is critical to understanding allopatric speciation. We examined behavioral isolation among four recently diverged allopatric species in the orangethroat darter clade (*Etheostoma*: *Ceasia*). We also examined behavioral isolation between each *Ceasia* species and the sympatric rainbow darter *Etheostoma caeruleum*. We asked (1) is behavioral isolation present between allopatric *Ceasia* species, and how does this compare to behavioral isolation with *E. caeruleum*, (2) does male color distance and/or genetic distance predict behavioral isolation between species, and (3) what are the relative contributions of female choice, male choice, and male competition to behavioral isolation? We found that behavioral isolation, genetic differentiation, and male color pattern differentiation were present between allopatric *Ceasia* species. Males, but not females, discerned between conspecific and heterospecific mates. Males also directed more aggression toward conspecific rival males. The high levels of behavioral isolation among *Ceasia* species showed no obvious pattern with genetic distance or male color distance. However, when the *E. caeruleum* was included in the analysis, an association between male aggression and male color distance was apparent. We discuss the possibility that reinforcement between *Ceasia* and *E. caeruleum* is driving behavioral isolation among allopatric *Ceasia* species.

Speciation requires the evolution of reproductive isolating barriers between taxa (Mayr 1995). A long‐standing goal in speciation research has been to identify the traits/behaviors contributing to reproductive isolation between taxa and the evolutionary forces giving rise to them. Comparative studies of speciation have considered the roles of time, sympatry versus allopatry, divergent ecological selection, and divergent sexual selection due to female choice (reviewed in Coyne and Orr 2004). The emerging consensus is that (a) reproductive isolating barriers increase across evolutionary time separating taxa (e.g., Sasa et al. 1998; Presgraves 2002; Price and Bouvier 2002; Fitzpatrick 2002; Russell 2003; Moyle et al. 2004), (b) differences in habitat/ecology are often associated with increased levels of reproductive isolation (e.g., Ryan 1990; Boughman 2002; Schluter and Price 1993; Fuller et al. 2005; Seehausen et al. 2008), (c) sympatric species pairs often have heightened reproductive isolation, presumably due to reinforcement (Coyne and Orr 1989, 1997), and (d) female mating preferences and prezygotic isolation often evolve early, particularly when species are sympatric (Gleason and Ritchie 1998; Turelli et al. 2001; Ritchie 2007). Hence, time since divergence, differences in ecology, reinforcement, and pronounced sexual selection via female mating preferences all favor enhanced reproductive isolation. Here, we consider the other side of the coin and ask how reproductive isolation evolves in recently diverged allopatric taxa that occupy similar environmental niches, and that (as of yet) lack evidence of female mating preferences. We ask whether discernible levels of reproductive isolation are present, which traits/behaviors predict reproductive isolation, and whether there is evidence that genetic distance (a surrogate for time since divergence) and/or sexual selection can account for the levels of reproductive isolation seen among allopatric taxa.

There are multiple reasons to expect that reproductive isolation should be low or absent among recently diverged allopatric taxa. First, recently diverged allopatric taxa may not have measurable reproductive isolation despite the fact that they differ in traits and/or genetic sequence. This is exemplified by the fact that hybrid swarms often occur when one species is introduced into the range of a close, allopatric relative (e.g., Wilde and Echelle 1992; Huxel 1999; Allendorf et al. 2001; Fitzpatrick et al. 2010). Second, species pairs that occur in similar habitats likely experience little divergent ecological selection, which should lower the likelihood of evolving isolating barriers (Martin and Mendelson 2012). Third, mating systems that are dominated by male–male competition and where sneakers frequently join spawning pairs may offer few opportunities for the evolution of male or female mate choice (Jones et al. 2001; Reichard et al. 2005). Hence, while sexual selection may be intense in such a system, there may be little reason to expect population divergence in preferences and target traits.

Here, we examined (a) whether behavioral isolation was present among four species of allopatric, recently diverged darters, (b) the relative roles of male and female behavior on behavioral isolation, and (c) whether genetic distance and/or color distance predicted behavioral isolation. Behavioral isolation occurs when mismatches in mating traits (signals and/or preferences) prevent mating between two species/populations. To deal with the problem of animals potentially mating indiscriminately in the laboratory, we also assayed behavioral isolation between each of the four species and a more distantly related sympatric darter species. Previous work on this system has shown behavioral isolation is almost complete between sympatric darter congeners (Zhou and Fuller 2014). The fact that these species are maintained in nature coupled with the fact that sympatric species are reluctant to hybridize in the laboratory provides some reassurance that animals are behaving as they would in a natural setting.

Darters are a highly diverse group of North American benthic stream fishes (Page 1983). Darter speciation appears to occur in allopatry, as the most closely related sister species do not co‐occur (Near and Benard 2004; Near et al. 2011). Within a given clade, darters often occupy similar environmental niches, suggesting that early divergence is not due to ecological selection (Schmidt 2009; Martin and Mendelson 2012, 2014). Instead, sexual selection is thought to play a pivotal role in darter speciation. Males of many species exhibit bright coloration or egg mimicry (Page 1983; Page and Burr 2011), and behavioral isolation evolves before larval F1 hybrid inviability (Mendelson 2003). Although many have assumed that male nuptial coloration is the target of female mating preferences (Mendelson 2003; Williams and Mendelson 2010, 2011; Williams et al. 2013), emerging evidence suggests that male coloration may function in aggressive signaling among males (Zhou et al. 2015; Zhou and Fuller 2016; Martin and Mendelson 2016).

The orangethroat darter clade (*Ceasia*) is well suited for studying the early stages of allopatric speciation. *Ceasia* consists of 15 recently diverged species that are all allopatric from one another (Ceas and Page 1997; Page and Burr 2011). A recent study by Bossu et al. (2013) reconstructed palaeodrainage connections in the eastern United States and built a time‐calibrated phylogenic tree to investigate the historical biogeography of the *Ceasia* clade. The *Ceasia* clade is estimated to have originated between 6.6 and 6.9 mya and to have diversified allopatrically (Bossu et al. 2013). Members of *Ceasia* were raised from the subspecies to species level due to differences in morphology and male coloration (Ceas and Page 1997), and a subsequent study has shown that there is genetic divergence between species (Bossu et al. 2013). However, prior to the present study, behavioral isolation had not been examined between any *Ceasia* species. Here we examined the evolution of behavioral isolation among four allopatric *Ceasia* species. We also compared levels of behavioral isolation among allopatric *Ceasia* species to levels of behavioral isolation between *Ceasia* and a more distantly related sympatric congener, *Etheostoma caeruleum* (rainbow darter). We examined the relationship between male color pattern divergence, genetic divergence, and three components of behavioral isolation: female choice among males, male choice among females, and male recognition of other males as competitors for females.

## Methods

### STUDY SPECIES, COLLECTION, AND MAINTENANCE

**Figure 1:**
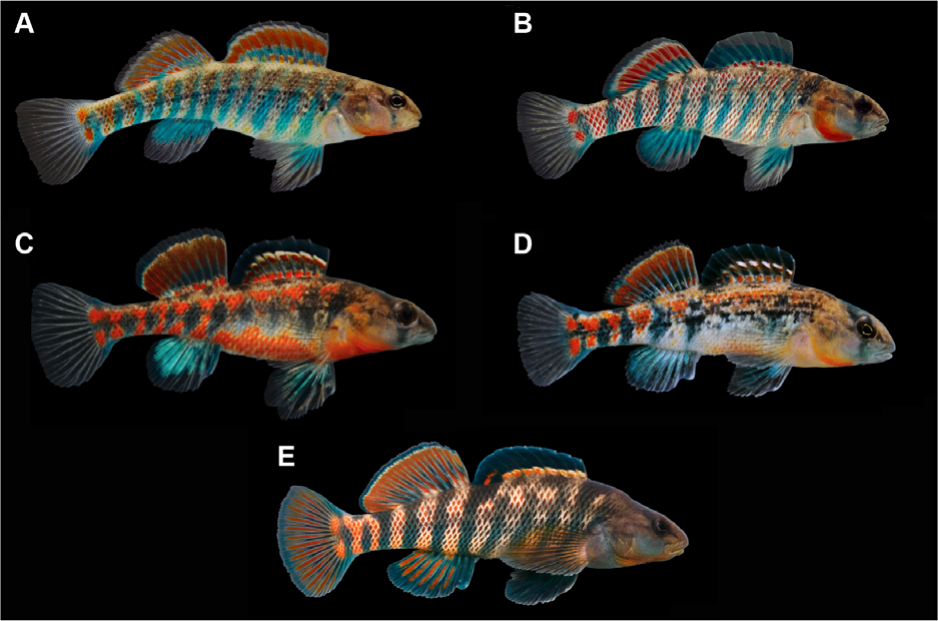
Males from each of the five species examined in this study: (A) *Etheostoma fragi*, (B) *E. uniporum*, (C) *E. burri*, (D) *E. spectabile*, and (E) *E. caeruleum*.

For our study, we used four allopatric species in the *Ceasia* clade: *Etheostoma fragi* (strawberry darter), *Etheostoma uniporum* (current darter), *Etheostoma burri* (brook darter), and *Etheostoma spectabile* (orangethroat darter), and a more distantly related, sympatric species, *E. caeruleum* (Fig. 1; Fig. S1). We originally used data from previous studies to choose pairs of *Ceasia* species that differed to varying degrees from one another in male color pattern and genetic sequence (i.e., low: *E. fragi* and *E. uniporum*; intermediate: *E. fragi* and *E. burri*; high: *E. fragi* and *E. spectabile*). We used the mitochondrial and nuclear gene phylogeny of Bossu et al. (2013) to initially select *Ceasia* species that varied in degree of relatedness, but we also measured genetic distance independently using Restriction site‐Associated DNA sequencing (RADseq) (see below). Likewise, we used images from field guides (Page 1983; Page and Burr 2011) and our own images to select *Ceasia* species that varied from one another in degree of color pattern similarity, but we also measured color distance between species with digital photography (see below).

Two populations of *E. caeruleum* were used, one from the Ozarks region and the other from Illinois (Table S1). The three *Ceasia* species from the Ozarks region were tested with the Ozarks *E. caeruleum*, and the *Ceasia* species from Illinois was tested with the Illinois *E. caeruleum*.

Adult fish were collected by kick‐seine in March 2015 (localities in Table S1). Both *Ceasia* and *E. caeruleum* were encountered at each site. Fish were transported back to the laboratory in aerated coolers. They were maintained in 38‐liter aquaria separated by species and sex at 20°C with a 13:11 light/dark cycle, and fed frozen bloodworms daily. Behavioral assays were performed prior to feeding on a given day.

### EXPERIMENTAL DESIGN FOR BEHAVIORAL ASSAYS

**Figure 2:**
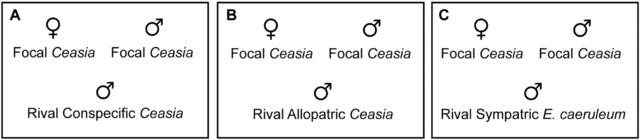
Experimental design for behavioral assays. A male and female *Ceasia* focal pair was used in three consecutive trial treatments in which the rival male was either (A) a conspecific *Ceasia*, (B) a heterospecific allopatric *Ceasia*, or (C) a sympatric *E. caeruleum*.

Our behavioral assays aimed to measure behavioral isolation between allopatric *Ceasia‐Ceasia* species pairs and between sympatric *Ceasia‐E. caeruleum* species pairs, and to determine the relative contributions of males and females to behavioral isolation. Behavioral assays were conducted from March through May 2015. Each trial took place in a 38 L aquarium with gravel substrate. To minimize disturbance, three sides of the observational tank were covered in black plastic. Each trial involved three fish: a *Ceasia* focal male, a *Ceasia* focal female, and a rival male (Fig. 2). Before each trial began, the focal male was placed in the observational tank and allowed to acclimate for 10 min. A conspecific focal female and a rival male were then placed into the tank with the focal male. When darters are first placed into a new tank, they typically respond by freezing and clamping their fins close to their bodies. We did not start a trial until all fish were freely swimming around the observational tank, indicating that they were acclimated. All darters acclimated quickly after being moved to an observational tank, and no fish took longer than 2 min to acclimate. After all three fish were acclimated, they were observed for 30 min. Each 30 min trial was divided into 30 s blocks. A focal male and focal female pair was observed together in three consecutive treatments that varied in the identity of the rival male. Rival males were either a conspecific *Ceasia* male, a heterospecific allopatric *Ceasia* male, or a heterospecific sympatric *E. caeruleum* male (Table 1, Fig. 2). Unique rival males were used, and the order of the three rival male treatments was randomized for each focal pair. We used rival males that were within 5 mm of the focal male's standard length. All focal females were gravid, discernible by distended abdomens.

**Table 1:** Each of the three species sets used in behavioral assays in forward (F) and reverse (R) direction

			Rival males		
Species set and direction	*n*	*Ceasia* Focal pair	Conspecific *Ceasia*	Allopatric *Ceasia*	Sympatric *E. caeruleum*
1F	8	*E. fragi*	*E. fragi*	*E. uniporum*	*E. caeruleum*
1R	8	*E. uniporum*	*E. uniporum*	*E. fragi*	*E. caeruleum*
2F	8	*E. fragi*	*E. fragi*	*E. burri*	*E. caeruleum*
2R	8	*E. burri*	*E. burri*	*E. fragi*	*E. caeruleum*
3F	8	*E. fragi*	*E. fragi*	*E. spectabile*	*E. caeruleum*
3R	8	*E. spectabile*	*E. spectabile*	*E. fragi*	*E. caeruleum* ^*^

*Eastern clade *E. caeruleum*. *E. caeruleum* in all other trial sets are from the Mississippi River Corridor clade.

Our behavioral assays were organized into three “sets,” each using *E. fragi* and one of the three other *Ceasia* species and *E. caeruleum* (Table 1). For each set, we performed behavioral assays where each *Ceasia* species (*E. fragi, E. uniporum, E. burri*, and *E. spectabile*) served as the focal male and female. We refer to these as the forward and reverse species sets (Table 1). In trials with *E. caeruleum, E. caeruleum* served as a rival male but was never a focal species. A total of eight replicates were conducted for each combination of species set, species set direction, and rival male treatment (3 species sets × 2 directions × 3 treatments × 8 replicates = 144 behavioral trials).

Male mate choice was measured for the rival males as male pursuit of the female. Male pursuit was measured as the proportion of 30 s blocks in which the rival male was within one body length of the female for at least five consecutive seconds (Zhou et al. 2015), divided by the total number of 30 s blocks in which either male was within one body length of the female for at least five consecutive seconds. Thus, we conducted no‐choice tests of male mate preference. Male aggression was measured as the number of fin flares and attacks performed by both the rival and focal male toward the other male during a trial (Zhou et al. 2015).

Female mate choice was measured as the relative proportion of nosedigs and headwags performed within one body length of the rival male. Nosedigs occur when a female jabs her snout into the substrate while searching for a suitable spawning location. Nosedigs are frequently used as a measure of female mating preference (Fuller 2003; Williams and Mendelson 2011). Females perform headwags when actively pursued by a male. Headwags signal receptivity to male courtship (Kozlowski 1979). We recorded the identity of the male(s) present within one body length for all nosedigs and headwags.

A trials was excluded from the analysis of headwags or nosedigs if a female did not perform the behavior in that trial. No trials were excluded from analyses of male behaviors, since at least one male in each trial performed female pursuit and aggressive behaviors. Table S2 lists sample sizes for each behavior.

### STATISTICAL ANALYSES OF BEHAVIORAL ASSAYS

For each of the three species sets, we used generalized linear models with a negative binomial distribution and log link function to analyze two measures of male aggression (i.e., number of fin flares and attacks) performed by the focal male and directed toward the rival male. Focal male species identity, rival male species identity (conspecific, heterospecific *Ceasia*, or *E. caeruleum*), and their interaction were the independent variables. This allowed us to examine whether focal males were more aggressive toward conspecific versus heterospecific rivals, and whether these effects were symmetrical for the forward and reverse trials. All statistical analyses were performed in R (version 3.2.1). Negative binomial generalized linear models were conducted using the glm.nb function in the package MASS (Venables and Ripley 2002). To examine pairwise differences among the rival male treatments, we performed post‐hoc tests using Tukey's multiple comparisons with the glht function in the package MULTCOMP (Hothorn et al. 2008). To consider the aggressive behavior of the rival male toward the focal male, we conducted two additional analyses following the same method used to analyze focal male aggressive behavior, but with rival male fin flares and rival male attacks serving as the dependent variables

For male mate choice, we performed a two‐way ANOVA with focal species identity, rival male identity, and the interaction terms as the independent variables. The dependent variable was the amount of time that the rival male pursued the focal female. This allowed us to test the prediction that rival *Ceasia* males would prefer to pursue conspecific over heterospecific females (Zhou et al. 2015). Likewise, *E. caeruleum* should have low levels of pursuit of *Ceasia* females. We conducted post‐hoc Bonferroni‐adjusted pairwise *t*‐tests to make pairwise comparisons among rival male treatments levels. We did not perform these analyses with focal *Ceasia* males as they were always with conspecific focal females.

Finally, we used ANCOVAs to asked whether females were more likely to respond to conspecific males compared to allopatric heterospecific *Ceasia* or sympatric *E. caeruleum* males. Previous work has shown that females spawn with the males that guard them (Zhou et al. 2015). Thus, we included male pursuit of female as a covariate in the analysis of nosedigs and in the analysis of headwags. For each of the three species sets, the full model included focal species, rival male identity, the interaction between focal species and rival male identity, and the proportion of time the focal female was guarded by the rival male versus the focal male.

### BEHAVIORAL ISOLATION INDICES

Behavioral data were used to estimate behavioral isolation indices following Martin and Mendelson (2016). Each index has a value between –1 to 1, where a positive value indicates more conspecific than heterospecific interactions were observed, a negative value indicates more heterospecific than conspecific interactions were observed, and a value of 0 indicates an equal number of conspecific and heterospecific interactions were observed (Stalker 1942; Mendelson 2003; Martin and Mendelson 2016). We calculated indices for female mate choice, male mate choice, and male aggression. Indices were calculated for each replicate within a set and then averaged across each species pair in a set.

To control for differences in the amount of time males spent pursuing females, the female choice index was calculated as the ratio of female nosedigs to the number of times a male attempted to pursue a female. The female mate choice index (FC) was calculated as:

$$FC=\frac{f_{c}}{p_{c}}-\frac{f_{h}}{p_{h}}$$

where f_c_ and f_h_ represent the number of nosedigs females performed near conspecific and heterospecific males, respectively. p_c_ and p_h_ represent the number of 30 s time blocks conspecific and heterospecific males spent in pursuit of the female during a trial, respectively.

The male mate choice index (MC) was calculated as:

$$MC=\frac{m_{c}-m_{h}}{m_{c}+m_{h}}$$

where m_c_ and m_h_ represent the proportion of time conspecific and heterospecific males spent pursuing the female during each trial.

The male–male aggression index (MA) was calculated as:

$$MA=\frac{a_{c}-a_{h}}{a_{c}+a_{h}}$$

where a_c_ and a_h_ represent the number of aggressive behaviors (i.e., chases and fin flares) performed between conspecific and heterospecific males.

### COLOR ANALYSIS

We used digital photography to quantify male coloration. We focused on components of male color pattern used in qualitative species diagnoses (Ceas and Page 1997). After each trial, we lightly anesthetized animals (0.01 g/L MS‐222 for 3 min). We then took photographs using a Nikon Coolpix D3300 digital camera under florescent lighting with the camera's factory setting for photography in florescent lighting. Each photograph contained a lateral view of an individual fish on a background of white 1 mm grid paper next to an X‐rite ColorChecker Mini Chart (Grand Rapids, MI). Inclusion of the color checker allows us to color correct digital images in Adobe Photoshop CS4 Extended using the inCamera 4.5 plug‐in (PictoColor Software, version 4.0.1), as described by Bergman and Beehner (2008).

For each species, digital photographs of 10 males were used in color analyses. Color analyses were conducted following the methodology outlined in Zhou et al. (2014). For each photograph, we took RGB measurements in Adobe Photoshop CS4 Extended using the Color Sampler Tool. For each fish, we took RGB measurements on both the red and the blue portions of the first dorsal fin, second dorsal fin, anal fin, and lateral bars. We also took RGB measurements on the throat and belly (which were always one solid color). Each RGB measurement gave separate values for R, G, and B. Average R, G, and B values were calculated from three replicate RGB measurements on the same photograph for each location on each fish. Thus, we obtained average R, G, and B values for 10 locations on each fish, for a total of 30 RGB variables.

We also measured the proportion of red and blue color on the first dorsal fin, second dorsal fin, anal fin, anterior body, and posterior body, for a total of 10 color proportion variables. Following Zhou et al. (2014), red and blue color proportions were measured in ImageJ (version 1.50c4) in CIE L^*^a^*^b^*^ color space. The perimeter of each body section was traced using the polygon selections tool in ImageJ, and the total number of pixels within each traced area was measured using the histogram tool. Red and blue proportions of each body area were calculated using the Threshold_Color ImageJ plugin (version 1.16, G. Landini; see Zhou et al. 2014 for full details).

Forty color variables (30 RGB and 10 color proportions) were collected from each male. We used the Mahalanobis distance to measure color distance between each species pair (Mahalanobis 1936). The Mahalanobis distance measures trait distances among groups by accounting for the variance and covariance within each group (Mahalanobis 1936; Arnegard et al. 2010; Martin and Mendelson 2014). The multivariate Mahalanobis distance is analogous to the univariate z‐score in that it removes the correlation between variables and standard. We calculated the squared Mahalanobis distance between each species pair with the pairwise.mahalanobis function of the HDMD package in R (version 3.2.1). We then took the square root of these values to calculate the interspecific Mahalanobis distance, referred to hereafter as male color distance.

### GENETIC DISTANCE

We used double digest RADseq to measure genetic distance among the five species. Nuclear DNA was extracted from 12 individuals from each species. Table S3 shows collection locations for individuals used in genetic analyses. Illumina libraries were prepared following Parchman et al. (2012). Nuclear DNA samples were digested with two restriction enzymes (EcoRI and Mse1) and barcoded for identification of individual samples. Samples were then pooled and amplified using 30 cycles of PCR. To obtain DNA fragments of a uniform size, the pooled PCR product was electrophoresed on a 2.5% agarose gel. Bands within the 500–600 bp range were excised and purified using a QIAquick Gel Extraction Kit (Qiagen). The pooled libraries were sequenced as 100 bp single‐end reads using an Illumina Hi‐Seq 2500 platform. We ran one lane of sequencing with 60 individuals total, which resulted in a mean coverage depth of 20X.

The Stacks software package (Catchen et al. 2011, 2013) was used to analyze the patterns of genetic structure. The program *process_radtags* was used to demultiplex samples and remove low quality reads (see Table S4). We used *ustacks* to build loci and call SNPs de novo for each individual, *cstacks* to compile a catalog of loci for each population, and *sstacks* to match each individual against the catalog. A minimum of three identical reads were required to infer a putative allele. We allowed a maximum of three mismatches when merging alleles into loci within an individual, and a maximum of two mismatches between loci when compiling the catalog of all RAD loci. These parameters resulted in a total catalog of 684,956 loci. We used the program *populations* to apply additional filters to the dataset and to conduct genetic analyses. Each locus was required to be present in every population and in at least 75% of the individuals within a population to be retained. Minor alleles present at lower than 0.04% were removed to control for false SNPs (i.e., sequencing errors). This filtering retained 18,295 loci. Of these, 17,162 were polymorphic and contained a total of 44,971 SNPs.

We used variant SNPs to calculate Nei's genetic distance (D_ST_; Nei 1972, 1978) and to conduct STRUCTURE and *K*‐means clustering analyses. The software packages used to conduct these analyses assume independence among SNPs. However, each locus in the catalog has the potential to contain multiple SNPs, which would be linked together on the same 100 bp RAD tag. To ensure only the first SNP was analyzed from each locus, we ran *populations* again with the same parameters as specified above but with the‐*–write_single_snp* option added. We also ran *populations* while excluding the outgroup, *E. caeruleum*, to obtain a *Ceasia*‐specific set of loci that would potentially allow for the detection of finer scale genetic differences among these species. When all five species were included, populations retained 16,968 variant loci. Excluding *E. caeruleum* resulted in *populations* retaining 19,896 variant loci.

We generated a GenePop (Rousset 2008) file in *populations* using the variant SNPs for all five species. We then imported the file into GenoDive (Version 2.0b27, Meirmans and van Tienderen 2004) and calculated Nei's standard genetic distance (D_ST_) between each species. We also performed a *K*‐means clustering analysis in GenoDive to obtain an estimate of the number of distinct genetic clusters (*K*). *K* was set to range from 1 through 8. We performed 20 repeats of the simulated annealing algorithm with 100,000 Markov Chain Monte Carlo (MCMC) steps. The optimal number of clusters was inferred from the *K* with the highest value for the pseudo‐*F* statistic (Caliński and Harabasz 1974; Meirmans 2012).

We also used STRUCTURE to determine the most likely value of *K*. We obtained two STRUCTURE (version 2.3.3, Pritchard et al. 2000) formatted output files from *populations* for the two datasets (with and without *E. caeruleum* included). Early STRUCTURE analyses revealed an F1 hybrid *E. caeruleum* x *E. uniporum* individual. This individual was excluded from all analyses. For all STRUCTURE analyses, we used 50,000 burn‐in steps with 150,000 MCMC steps. Ranges for *K* were set to 1 through 8 when all five species were included, and 1 through 7 when *E. caeruleum* was excluded. Analyses for each potential value of *K* were run 50 times. The true number of genetic clusters present for each dataset was determined using the Delta *K* method (Evanno et al. 2005). Delta *K* values were calculated using Structure Harvester (Earl and vonHoldt 2012).

### RELATIONSHIP BETWEEN BEHAVIORAL ISOLATION, COLOR DISTANCE, AND GENETIC DISTANCE

**Figure 3:**
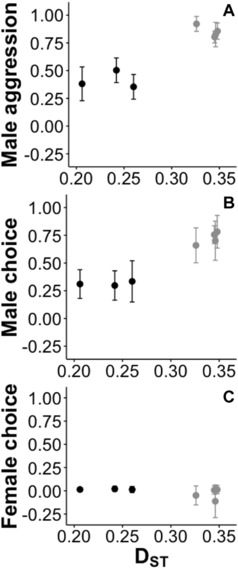
Behavioral isolation indices with 95% confidence intervals for (A) male aggression, (B) male choice, and (C) female choice versus Nei's genetic distance (D_ST_). Each point represents an individual pairwise species comparison. *Ceasia–Ceasia* comparisons are shown in black and *Ceasia‐E. caeruleum* comparisons are shown in gray.

**Figure 4:**
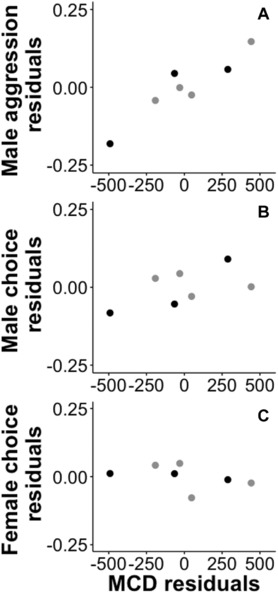
Behavioral isolation indices for (A) male aggression, (B) male choice, and (C) female choice versus male color distance (MCD). Each point represents an individual pairwise species comparison. *Ceasia–Ceasia* comparisons are shown in black and *Ceasia‐E. caeruleum* comparisons are shown in gray.

To examine the relationship between behavioral isolation and genetic distance, we plotted the three behavioral isolation indices (male choice, male aggression, and female choice) with 95% confidence intervals versus pairwise D_ST_ values (Fig. 3). We also examined the relationship between behavioral isolation and male color distance. To control for the potential influence of genetic distance on these variables, each of the three indices of behavioral isolation and male color distance were regressed onto D_ST_. We then plotted the residuals of these analyses against one another (Fig. 4). We visually examined the plots of behavioral isolation versus D_ST_ (Fig. 3) and behavioral isolation versus male color distance (Fig. 4) to determine whether any trends existed among the three *Ceasia‐Ceasia* species comparisons and among the four *Ceasia*‐*E. caeruleum* comparisons. Phylogenetically independent contrasts (Felsenstein 1985) were not feasible due to the number of independent species pairs examined.

## Results

### DO MALES DISCERN CONSPECIFIC FROM HETEROSPECIFIC MALE RIVALS?

Focal male *Ceasia* were more aggressive toward conspecific than heterospecific rivals, indicating that they could discriminate males of closely related species (Table 2). Aggression was lowest toward the more distantly related *E. caeruleum*, and was intermediate toward heterospecific allopatric *Ceasia* males. The results were most striking for fin flares. Across all three species sets, focal males performed 15X more fin flares toward conspecific males compared to *E. caeruleum* males (Figs. S2–S4). In one of the three species sets (*E. fragi—E. uniporum—E. caeruleum*), focal males performed significantly more fin flares toward conspecific than heterospecific *Ceasia*. The same general pattern was observed for attacks, but focal males performed significantly more attacks toward conspecific than heterospecific *Ceasia* only in the *E. fragi—E. burri—E. caeruleum* species set. This same set was notable because the two focal species differed in aggression. Focal male *E. burri* performed 5 × more attacks on both conspecific *Ceasia* and allopatric heterospecific *Ceasia* rivals compared to focal male *E. fragi* (Table 2, Fig. S3).

**Table 2:** Negative binomial regression on focal male behavior toward rival males

A. *E. fragi* – *E. uniporum – E. caeruleum* (1F and 1R)			
	df	Test statistic	*P*
Variable: Focal male fin flares			
Rival male identity	2	34.652	<0.00001
Conspecific versus allopatric *Ceasia*		–2.980	<0.01
Conspecific versus sympatric *E. caeruleum*		–5.533	<0.001
Focal male identity	1	0.436	0.509
Rival male identity × focal male identity	2	3.320	0.190
Variable: Focal male attacks			
Rival male identity	2	13.933	<0.001
Conspecific versus allopatric *Ceasia*		–1.316	0.382
Conspecific versus sympatric *E. caeruleum*		–3.535	<0.01
Focal male identity	1	1.043	0.307
Rival male identity × focal male identity	2	0.620	0.734
B. *E. fragi – E. burri – E. caeruleum* (2F and 2R)			
Variable: Focal male fin flares			
Rival male identity	2	28.791	<0.00001
Conspecific versus allopatric *Ceasia*		–2.073	0.094
Conspecific versus sympatric *E. caeruleum*		–5.282	<0.001
Focal male identity	1	0.163	0.687
Rival male identity × focal male identity	2	0.447	0.800
Variable: Focal male attacks			
Rival male identity	2	25.747	<0.00001
Conspecific versus allopatric *Ceasia*		–2.693	<0.05
Conspecific versus sympatric *E. caeruleum*		–4.896	<0.001
Focal male identity	1	12.740	<0.001
Rival male identity × focal male identity	2	0.733	0.693
C. *E. fragi – E. spectabile – E. caeruleum* (3F and 3R)			
Variable: Focal male fin flares			
Rival male identity	2	24.370	<0.0001
Conspecific versus allopatric *Ceasia*		–0.839	0.677
Conspecific versus sympatric *E. caeruleum*		–4.748	<0.001
Focal male identity	1	0.331	0.565
Rival male identity × focal male identity	2	0.731	0.694
Variable: Focal male attacks			
Rival male identity	2	12.447	<0.01
Conspecific versus allopatric *Ceasia*		–0.955	0.60
Conspecific versus sympatric *E. caeruleum*		–3.175	<0.01
Focal male identity	1	0.142	0.707
Rival male identity × focal male identity	2	0.863	0.649

Posthoc comparisons using Tukey's test for multiple contrasts are shown for significant effects of rival male identity. The table headings (A–C) list the two *Ceasia* species in the species set (*E. fragi* and a heterospecific allopatric *Ceasia* species) followed by the sympatric, distantly related *E. caeruleum*.

**Figure 5:**
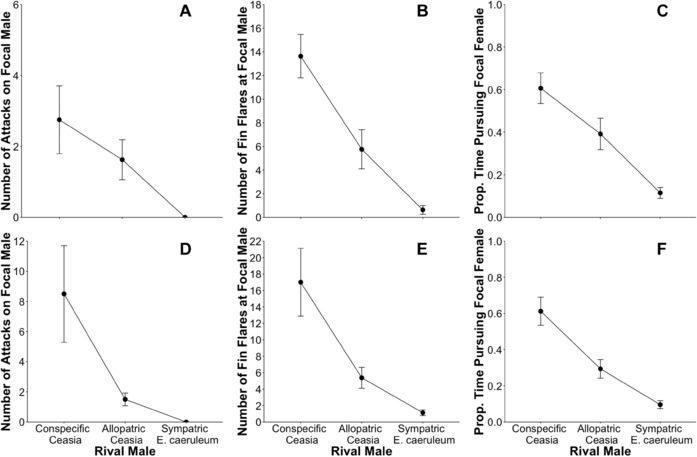
Rival male behavior toward focal males and focal females. (A–C) Species set 1F with *E. fragi* as the focal pair and conspecific *Ceasia* rival male, and *E. uniporum* as the allopatric *Ceasia* rival male. (D–F) Species set 1R with *E. uniporum* as the focal pair and conspecific *Ceasia* rival male, and *E. fragi* as the allopatric *Ceasia* rival male. (A, D) Rival male attacks on focal male. (B, E) Rival male fin flares at focal male. (C, F) Rival male pursuit of focal female.

We observed similar patterns of increased aggression toward conspecifics over heterospecific males in rival males. Conspecific rival males were most aggressive, *E. caeruleum* rival males were least aggressive, and heterospecific *Ceasia* rival males were intermediate (Figs. 5, S5, and S6). Hence, there were high levels of species discrimination between heterospecific *Ceasia* males even though they are allopatric. Across all three species sets, the numbers of fin flares and the number of attacks directed at the focal male differed as a function of rival male identity (Table 3).

**Table 3:** Negative binomial regression on rival male behavior towards focal male

A. *E. fragi* – *E. uniporum – E. caeruleum* (1F and 1R)			
	df	Test statistic	*P*
Variable: Rival male fin flares			
Rival male identity	2	47.927	<0.00001
Conspecific versus allopatric *Ceasia*		–2.652	<0.05
Conspecific versus sympatric *E. caeruleum*		–5.782	<0.001
Focal male identity	1	0.535	0.465
Rival male identity × focal male identity	2	0.957	0.620
Variable: Rival male attacks			
Rival male identity	2	21.186	<0.00001
Conspecific versus allopatric *Ceasia*		–1.048	0.502
Conspecific versus sympatric *E. caeruleum*		–0.004	1.000
Focal male identity	1	6.704	<0.01
Rival male identity × focal male identity	2	3.046	0.218
B. *E. fragi – E. burri – E. caeruleum* (2F and 2R)			
Variable: Rival male fin flares			
Rival male identity	2	43.896	<0.00001
Conspecific versus allopatric *Ceasia*		–2.054	0.096
Conspecific versus sympatric *E. caeruleum*		–5.783	<0.001
Focal male identity	1	0.235	0.628
Rival male identity × focal male identity	2	0.938	0.626
Variable: Rival male attacks			
Rival male identity	2	28.131	<0.00001
Conspecific versus allopatric *Ceasia*		–1.773	0.167
Conspecific versus sympatric *E. caeruleum*		1.093	<0.001
Focal male identity	1	3.119	0.077
Rival male identity × focal male identity	2	5.586	0.061
C. *E. fragi – E. spectabile – E. caeruleum* (3F and 3R)			
Variable: Rival male fin flares			
Rival male identity	2	26.649	<0.00001
Conspecific versus allopatric *Ceasia*		–5.005	0.091
Conspecific versus sympatric *E. caeruleum*		–2.088	<0.001
Focal male identity	1	0.547	0.460
Rival male identity × focal male identity	2	0.064	0.969
Variable: Rival male attacks			
Rival male identity	2	31.270	<0.00001
Conspecific versus allopatric *Ceasia*		–2.473	<0.05
Conspecific versus sympatric *E. caeruleum*		–0.004	1.000
Focal male identity	1	0.160	0.689
Rival male identity × focal male identity	2	0.496	0.780

Posthoc comparisons using Tukey's test for multiple contrasts are shown for significant effects of rival male identity. The table headings (A–C) list the two *Ceasia* species in the species set (*E. fragi* and a heterospecific allopatric *Ceasia* species) followed by the sympatric, distantly related *E. caeruleum*.

### DO MALES DISCERN BETWEEN CONSPECIFIC AND HETEROSPECIFIC FEMALES?

Rival males clearly altered their pursuit behavior depending on whether females were conspecific or heterospecific. Conspecific *Ceasia* rival males spent the most time pursuing the focal female; heterospecific *Ceasia* rivals were intermediate in focal female pursuit. Sympatric *E. caeruleum* rival males spent little time pursuing the focal female (Figs. 5C,F, S5C,F, and S6C,F). On average, the amount of time spent in pursuit of the focal *Ceasia* female was 5 × greater for conspecific *Ceasia* rival males compared to heterospecific *E. caeruleum* rival males (Figs. 5C,F, S5C,F and S6C,F). These differences between conspecific *Ceasia* versus heterospecific *E. caeruleum* were significant in all three trial sets (Table 4). Across all trial sets, conspecific *Ceasia* rival males spent 2X more time spent pursuing the *Ceasia* focal females compared to heterospecific *Ceasia* rival males. In two of the three species sets, these differences were statistically significant (Table 4B,C).

**Table 4:** ANOVA on rival male behavior toward focal female

A. *E. fragi* – *E. uniporum – E. caeruleum* (1F and 1R)			
	df	Test statistic	*P*
Variable: Rival male pursuit of focal female			
Rival male identity	2.42	10.054	<0.001
Conspecific versus allopatric *Ceasia*	45	–1.5139	0.4112
Conspecific versus sympatric *E. caeruleum*	45	–5.9158	<0.00001
Focal pair identity	1.42	0.0153	0.9020
Rival male identity × focal pair identity	2.42	0.6469	0.5288
B. *E. fragi* – *E. burri – E. caeruleum* (2F and 2R)			
Variable: Rival male pursuit of focal female			
Rival male identity	2.42	13.606	<0.00001
Conspecific versus allopatric *Ceasia*	45	–3.2371	<0.01
Conspecific versus sympatric *E. caeruleum*	45	–8.6079	<0.00001
Focal pair identity	1.42	2.8817	0.0970
Rival male identity × focal pair identity	2.42	1.1867	0.3153
C. *E. fragi* – *E. spectabile – E. caeruleum* (3F and 3R)			
Variable: Rival male pursuit of focal female			
Rival male identity	2.42	5.3156	<0.01
Conspecific versus allopatric *Ceasia*	45	–2.6836	<0.01
Conspecific versus sympatric *E. caeruleum*	45	–5.1759	<0.000001
Focal pair identity	1.42	0.5853	0.4485
Rival male identity × focal pair identity	2.42	0.5790	0.5649

Posthoc Bonferroni‐adjusted pairwise *t*‐tests are shown for significant effects of rival male identity. The table headings (A–C) list the two *Ceasia* species in the species set (*E. fragi* and another a heterospecific allopatric *Ceasia* species) followed by the sympatric, distantly related *E. caeruleum*.

### DO FEMALES DISCERN BETWEEN CONSPECIFIC AND HETEROSPECIFIC MALES?

We found no evidence for female mate preference for conspecifics over heterospecifics. The number of nosedigs and headwags performed toward males did not differ among rival males when rival male pursuit was included as a covariate in the analysis (Figs. S7–S9, Table S5. Hence, there is no evidence that females adjusted their willingness to spawn due to the identity of the male that was guarding her.

### BEHAVIORAL ISOLATION INDICES

Behavioral isolation was high for male mate choice and for male aggression, but was low for female mate choice (Table 5). For all *Ceasia* species pairs, indices of male choice and male aggression were positive and greater than zero (male choice: *t* = 6.50, df = 6, *P* < 0.001; male aggression: *t* = 7.27, df = 6, *P* < 0.001), indicating a behavioral preference for responding to conspecifics over heterospecifics. Male choice and male aggression indices were twice as high for *Ceasia*—*E. caeruleum* pairings compared to heterospecific *Ceasia* pairings. Female choice indices did not differ significantly from zero (*t* = –0.69, df = 6, *P* = 0.51), indicating females show little preference for conspecific over heterospecific males.

**Table 5:** Behavioral isolation indices for male choice (MC), male aggression (MA), and female choice (FC), male color distance (MCD), and Nei's standard genetic distance (D_ST_)

Species pair	MC	MA	FC	MCD	D_ST_
*E. fragi–E. uniporum*	0.31 ± 0.07	0.38 ± 0.08	0.01 ± 0.01	457.628	0.206
*E. fragi–E. burri*	0.30 ± 0.07	0.50 ± 0.06	0.02 ± 0.01	547.442	0.242
*E. fragi–E. spectabile*	0.34 ± 0.10	0.35 ± 0.06	0.01 ± 0.02	341.987	0.260
*E. fragi–E. caeruleum*	0.76 ± 0.06	0.80 ± 0.05	0.01 ± 0.04	1685.93	0.345
*E. uniporum–E. caeruleum*	0.70 ± 0.09	0.82 ± 0.06	–0.11 ± 0.13	1937.85	0.346
*E. burri–E. caeruleum*	0.66 ± 0.08	0.92 ± 0.03	–0.05 ± 0.05	2086.53	0.326
*E. spectabile–E. caeruleum*	0.78 ± 0.08	0.86 ± 0.04	0.01 ± 0.02	1884.18	0.348

For each species pair the *Ceasia* species that was used as the focal pair is listed first, followed by the species that was used for the rival male (heterospecific *Ceasia* or *E. caeruleum*). Behavioral isolation indices are shown as mean ± standard error.

### AMONG SPECIES PATTERNS IN GENETIC DISTANCE

As with our behavioral isolation assays, our genetic analysis indicates that all five species were distinct evolutionary units; all four *Ceasia* species differed significantly from one another, and *E. caeruleum* was an obvious genetic outgroup to *Ceasia*. One clear F1 hybrid between *E. uniporum* and *E. caeruleum* was detected, but this individual was excluded from the analysis. Table 6 shows the population genetic statistics for the total loci retained (both variant and invariant) and the variant loci alone. As expected, *E. caeruleum* had the largest number of private alleles. In general, *E. caeruleum* also harbored greater genetic variation than the *Ceasia* species; the observed heterozygosity, nucleotide diversity, and percent polymorphic loci were highest in *E. caeruleum*. Although these indices of genetic variation were nearly as high in *E. uniporum* as they were in *E. caeruleum*, the observed heterozygosity, nucleotide diversity, and percent polymorphic loci across all loci in *E. caeruleum* were between 1.5 and 3X higher than that present in *E. fragi*, *E. burri*, and *E. spectabile*.

**Table 6:** Population genetic statistics for the four allopatric *Ceasia* species (*E. fragi*, *E. uniporum*, *E. burri*, and *E. spectabile*) and the sympatric *E. caeruleum*

Species	Private alleles	% Poly	All loci P	Variant loci P	All loci H_obs_	Variant loci H_obs_	All loci π	Variant loci π
*E. fragi*	7352	0.2167	0.9994	0.9778	0.0298	0.0008	0.0308	0.0008
*E. uniporum*	8178	0.2936	0.9991	0.9686	0.0401	0.0011	0.0432	0.0012
*E. burri*	4531	0.2334	0.9993	0.9750	0.0339	0.0009	0.0338	0.0009
*E. spectabile*	4417	0.1139	0.9997	0.9891	0.0147	0.0004	0.0151	0.0004
*E. caeruleum*	12,392	0.3396	0.9991	0.9667	0.0417	0.0011	0.0463	0.0013

Statistics are shown for the 18,295 fixed and variant loci (all loci) and for the 17,162 variant loci. Statistics were calculated in Stacks (Catchen et al. 2011, 2013). % Poly, percent polymorphic loci; *P*, average major allele frequency; H_obs_, observed heterozygosity; π, nucleotide diversity.

Pairwise D_ST_ values for *Ceasia‐Ceasia* and *Ceasia‐E. caeruleum* species pairs differed significantly from one another (Table 5; *t* = –6.31, df = 2.42, *P* < 0.05). The highest D_ST_ value was 0.348 between *E. spectabile* and *E. caeruleum* and the lowest was 0.206 between *E. fragi* and *E. uniporum*. The D_ST_ values for *Ceasia‐Ceasia* species pairs ranged from 0.206 to 0.260. The D_ST_ values for *Ceasia*‐*E. caeruleum* species pairs ranged from 0.326 to 0.348. All D_ST_ values differed from zero (*t* = 13.30, df = 6, *P* < 0.0001).

STRUCTURE identified two main clusters when *E. caeruleum* was included in the analysis. One cluster corresponded to *E. caeruleum*, a second to the four *Ceasia* species (Tables S6–S7; Fig. S10A). When *E. caeruleum* was excluded, STRUCTURE identified two main clusters within *Ceasia*. *E. burri* and *E. spectabile* were grouped together into one cluster, and *E. fragi* and *E. uniporum* were grouped together into a second cluster (Tables S8–S9; Fig. S10B).

While STRUCTURE did not detect the four *Ceasia* species as distinct groups, these species were recovered via *K*‐means clustering. When all five species were included, *K*‐means clustering identified each species as a distinct cluster, with an optimal *K* of 5 (Table S10).

### AMONG SPECIES PATTERNS IN COLOR DISTANCE

Analyses of male color distance also revealed significant differences between species. All five species differed from one another in male color pattern (Table 5; male color distance > 0 for all species pairs; *t* = 4.30, df = 6, *P* < 0.01). Differences in male color distance were larger for *Ceasia‐E. caeruleum* than for *Ceasia‐Ceasia* species pairs (*t* = 14.22, df = 4.93, *P* < 0.0001). Within *Ceasia*, genetic distance was not related to male color distance. *E. fragi* and *E. spectabile* had the lowest male color distance, despite having the largest pairwise genetic distance within *Ceasia*. Conversely, *E. fragi* and *E. uniporum* had the lowest pairwise genetic distance within *Ceasia*, yet they exhibited an intermediate male color distance.

### DO GENETIC DIFFERENCES AND/OR COLOR DIFFERENCES PREDICT BEHAVIORAL ISOLATION?

Male components of behavioral isolation were higher among the *Ceasia‐E. caeruleum* comparisons than in the *Ceasia‐Ceasia* comparisons and these patterns coincide with large differences in genetic distance (Fig. 3) and male color distance (Fig. 4). Although there were high levels of behavioral isolation (i.e., male choice and male aggression) between *Ceasia* species, there were no obvious correlations with genetic distance (Fig. 3A,B). Behavioral isolation values did not vary among the three *Ceasia‐Ceasia* comparisons or among the four *Ceasia‐E. caeruleum* comparisons, as evidenced by their 95% confidence intervals.

We did not have enough phylogenetically independent species pairs to utilize a phylogenetically controlled regression of behavioral isolation on male color distance. We performed a regression on the raw data and calculated the residuals of male color distance as a function of genetic distance and the residuals of each component of behavioral isolation as a function of genetic distance. We subsequently regressed the behavioral isolation residuals onto the male color distance residuals. This analysis showed that male color distance residuals predicted male aggression residuals (*R* ^2^ = 0.87, F_1,5_ = 33.65, *P* = 0.002; Fig. 4A). This indicates that species pairs with greater differences in coloration were less likely to fight, since a larger male aggression index value represents a larger preference for fighting with conspecifics over heterospecifics. Male color distance did not predict male choice residuals (*R* ^2^ = 0.32, F_1,5_ = 2.38, *P* = 0.18; Fig. 4B) or female choice residuals (*R* ^2^ = 0.18, F_1,5_ = 1.07, *P* = 0.35; Fig. 4C).

## Discussion

Three main results emerged from this study. First, behavioral isolation among taxa was created by male preferences for conspecific over heterospecific females, whereas female mating preferences for conspecific males were absent. Second, males also discerned between conspecific and heterospecific males, preferentially directing aggression toward conspecifics. Additionally, male color distance was associated with the ability of males to discern conspecific (vs heterospecific) male rivals. Third, we showed high levels of behavioral isolation among recently diverged, allopatric *Ceasia* species, yet we were unable to explain how this behavioral isolation evolved; no patterns within *Ceasia* emerged between behavioral isolation, genetic distance, and male color distance. We discuss the implications of these results below.

### THE RELATIVE IMPORTANCE OF MALE VERSUS FEMALE BEHAVIOR ON REPRODUCTIVE ISOLATION

Male darters often show bright, conspicuous coloration that varies among species. This pattern has led to the hypothesis that these colors are important to female mating preferences and reproductive isolation (Williams and Mendelson 2010, 2011; Williams et al. 2013). Yet, here we showed that male mate choice plays a critical role in behavioral isolation. Males of all four species of *Ceasia* discriminated against heterospecific *Ceasia* and *E. caeruleum* females. Hence, males can distinguish between conspecific and heterospecific mates, even at relatively early stages of allopatric divergence. Conversely, female *Ceasia* did not express mate preferences for conspecifics. The lack of female discrimination against heterospecific males is in keeping with numerous other studies on this system that have consistently found no evidence for female mate choice at either the within or among species levels (Pyron 1995; Fuller 2003; Zhou et al. 2015). Instead, there is strong evidence for male mate choice among females (Zhou et al. 2015).

Theoretical and empirical studies of speciation via sexual selection have focused largely on the evolution of female mating preferences (reviewed in Panhuis et al. 2001), with less attention given to the roles of males. The assumption is that females have a larger cost associated with reproduction and experience strong selection to choose high‐quality mates (Bateman 1948; Trivers 1972). However, males can also have a significant cost associated with mating that may favor male choice (reviewed in Edward and Chapman 2011; Qvarnström et al. 2012). Male choice need not be limited to systems with reversed sex roles or male parental care. Investment in secondary sex traits (either to attract mates or compete with rivals) can increase male mating costs via increased mortality rates (Kokko and Monaghan 2001). In darters, males engage in frequent, prolonged bouts of competition over access to females, decrease their foraging rates on the spawning grounds, and can potentially become injured while fighting. In addition, choosiness may be beneficial in darters because mistakenly mating with more distantly related sympatric heterospecifics can result in reduced hybrid viability (Zhou 2014; R. L. Moran unpubl. data). The cost of male choice coupled with the benefit of choosiness may favor male discrimination between conspecific and heterospecific females in darters.

The lack of female mating preferences in *Ceasia* is notable given that males are so colorful, and that coloration varies among males even within populations (Zhou et al. 2014). We suspect that female mating preferences are costly in darters for three reasons. First, prolonged female choice that delays spawning may reduce egg viability. In many externally fertilizing fish, egg viability decreases with time since ovulation (McEvoy 1984; Formacion et al. 1993; Bromage et al. 1994; de Gaudemar and Beall 1998), and preliminary data indicate that this is the case in darters (in prep.). The optimal strategy for females may be to spawn quickly after ovulation. Second, females may lack the ability to exert mating preferences. Males congregate on gravel riffles where spawning occurs. When ready to spawn, females move to the riffles and are quickly pursued by many males. Females bury themselves in the gravel and wait for a male to initiate spawning. The female cannot see which male has initiated spawning as she is buried in the gravel. Instead, the female spawns with the first male to initiate spawning (Pyron 1995). Third, spawning pairs are often joined by other males acting as sneakers, precluding female choice (Fuller 1999). These three properties–‐a rapid decline in egg viability following ovulation, an inability to identify the male that initiates spawning, and high levels of sneaker mating–‐may make female choice costly relative to its benefits. Similar dynamics occur in other external fertilizers (Warner and Robertson 1978; Warner 1987).

Darter species have traditionally been diagnosed using differences in male nuptial ornamentation. Yet our behavioral results suggest that species‐diagnostic, female traits are present and that the levels of diversity rival those observed in male sex traits. We doubt that these are visual cues (but see Williams and Mendelson 2010, 2011; Ciccotto et al. 2013). Many darters lack distinguishing female coloration or morphological traits, especially at the within‐ subgenus level (Page and Burr 2011). In addition, males that come across a heterospecific female already buried in the gravel (and thus with any potential visual cues hidden) often fail to spawn with the female (R. L. Moran pers. obs.). This suggests that males use olfactory cues. Several species of darters, including *Ceasia* and *E. caeruleum*, respond to chemical alarm cues from conspecifics and some heterospecifics (Smith 1979; Commens and Mathis 1999; Haney et al. 2001). There is also pronounced variation in olfactory system morphology among darters (Ceas and Page 1997; Page and Burr 2011). Hence, darters may potentially join the ranks of taxa demonstrating large effects of olfaction on species recognition (reviewed in Ache and Young 2005).

Finally, we note that the mating dynamics in *Ceasia* and *E. caeruleum* stand in contrast to those in snubnose darters. Studies examining female mate choice in snubnose darters and its allies have found mixed support for female mate choice depending on whether comparisons were made between sympatric or allopatric species. Female snubnose darters discriminate against sympatric males (Williams and Mendelson 2010, 2011), but do not discriminate against males from closely related allopatric species (Martin and Mendelson 2016). Instead, like our findings in *Ceasia*, male snubnose darters discriminate against allopatric heterospecific females and males (Martin and Mendelson 2016).

### THE ROLE OF MALE COMPETITION AND MALE COLOR PATTERN

There is strong evidence that male coloration is used by male darters to signal both species identity and competitive ability (Zhou et al. 2015; Zhou and Fuller 2016; Martin and Mendelson 2016). Previous work has shown that within species, male color pattern predicts male reproductive success via ability to guard a female from other males and secure spawnings (Zhou et al. 2015). Furthermore, altering the lighting environment impairs the ability of males to see the red components of the color pattern and decreases aggressive response toward conspecific males (Zhou and Fuller 2016).

We found that male *Ceasia* discerned conspecific male rivals from closely related *Ceasia* males and from *E. caeruleum* males. Additionally, the residuals of male color distance (corrected for genetic distance) predicted behavioral isolation via male aggression residuals (corrected for genetic distance). Hence, species pairs that had higher than expected differences in male color pattern were less likely to engage in male–male competition. The same effects were not found for male mate choice or female mate choice. In some systems (anoles and cichlids), male color pattern is under selection from female mate choice in addition to male competition (Macedonia and Stamps 1994; Seehausen and Schluter 2004; Pauers et al. 2008). Darters are unique in that an elaborate male signal has evolved due to male–male competition without functioning in the context of female choice, and is utilized by males in species recognition.

### THE DRIVERS OF REPRODUCTIVE ISOLATION

We observed surprisingly high levels of behavioral isolation among newly diverged, allopatric species of *Ceasia*. These species were originally described based on qualitative descriptions of variation in male coloration (Ceas and Page 1997). Bossu et al. (2013) subsequently created a phylogeny using two mitochondrial genes and 10 nuclear genes. Here, we used RADseq and digital photography and showed that there is, indeed, significant variation in male coloration and genetic distance among species. The patterns of relatedness that we observed largely reflect those shown previously; *E. fragi* is more closely related to *E. uniporum* than it is to either *E. burri* or *E. spectabile*, and *Ceasia* species are more closely related to one another than they are to *E. caeruleum*. Furthermore, the lower levels of allelic variation present within *Ceasia* compared to *E. caeruleum* reflect the biology of this system. *Ceasia* species are typically restricted to small headwater streams, resulting in low levels of gene flow among populations (Echelle et al. 1975, 1976). In contrast, *E. caeruleum* can be found in larger order streams and rivers, allowing for higher levels of gene flow among populations.

The high levels of male‐driven behavioral isolation observed among *Ceasia* species was unexpected. Many closely related, allopatric species will readily hybridize upon secondary contact–whether it be in nature or in the laboratory (e.g., Pinceel et al. 2005; Gay et al. 2007; Harper and Hart 2007). Furthermore, males were presented with a no‐choice situation in which they could only choose whether or not to pursue the female. No‐choice mating assays are thought to underestimate levels of behavioral isolation (Foote and Larkin 1988; Verrell 1990; Coyne 1993; Hatfield and Schluter 1996). How these high levels of behavioral isolation evolved among recently diverged, allopatric taxa is unclear. There is no support for the idea that genetic distance or male color pattern distance accounts for behavioral isolation within *Ceasia*. One possibility is that the *Ceasia* species pairs we examined were too similar in genetic distance to detect a meaningful signature. Additionally, the number of within‐*Ceasia* species pairings analyzed here is admittedly low.

Clearly, behavioral isolation is higher between *Ceasia* and *E. caeruleum* than it is within *Ceasia*, but these two groups differ in multiple aspects. Both genetic distance and male color pattern distance is higher in *Ceasia‐E. caeruleum* species pairs compared to *Ceasia‐Ceasia* species pairs. Perhaps more important is the fact that *Ceasia* and *E. caeruleum* occur in sympatry and likely experience reinforcement. Previous work shows a pattern consistent with reproductive character displacement (RCD) between *Ceasia* and *E. caeruleum*, with preferences for conspecifics heightened in sympatry (Zhou and Fuller 2014). Hybridization occurs between *Ceasia* and *E. caeruleum* in nature (Ray et al. 2008; Keck and Near 2009; Bossu and Near 2009; this study), and postzygotic isolation is present (Zhou 2014; R. L. Moran unpubl. data). These observations are consistent with reinforcement (Servedio and Noor 2003; Coyne and Orr 2004).

The presence of reinforcement in this system may also explain why males bias their aggression toward conspecific males. Increased male discrimination against heterospecific females in sympatry via reinforcement may incidentally increase the costs associated with heterospecific male fighting. This can potentially favor increased male discrimination against heterospecific males in sympatry, that is agonistic character displacement (ACD; Grether et al. 2009; Qvarnström et al. 2012). Our working hypothesis is that (a) male–male aggression is very costly and (b) males are more likely to escalate aggression when fighting over conspecific females. This creates a positive feedback where selection further favors increased levels of recognition for both conspecific (vs heterospecific) females and conspecific (vs heterospecific) males. These high levels of discrimination may, ironically, allow for *Ceasia* and *E. caeruleum* to occur in very close sympatry (i.e., on the same riffles), increasing their potential to hybridize, and further fueling reinforcement (Vallin et al. 2012).

Another untested hypothesis is that cascade reinforcement has caused heightened behavioral isolation among these allopatric species (reviewed in Ortiz‐Barrientos et al. 2009), leading to a pattern of cascade RCD and cascade ACD within *Ceasia*. Cascade reinforcement could occur if reinforcement between *E. caeruleum* and *Ceasia* results in either heightened preferences for conspecifics or radically altered target traits such that allopatric *Ceasia* no longer recognize one another as potential mates. Theoretical studies of cascade reinforcement suggest that it is particularly likely to occur in species with low gene flow (reviewed in Comeault and Matute 2016), such as these headwater species of darters. Obviously, the data presented here do not allow us to test this hypothesis as all of the *Ceasia* species were sympatric with *E. caeruleum*. The critical test is whether *Ceasia* that are allopatric to *E. caeruleum* have lower behavioral isolation than *Ceasia* that are sympatric with *E. caeruleum*. Preliminary evidence indicates that this may be the case (Moran and Fuller in review).

In conclusion, this study found that recently diverged allopatric *Ceasia* have surprisingly high levels of behavioral isolation that is created by male mate choice and male recognition of rival males. Female mate choice was absent. Neither genetic distance nor male color pattern distance account for the levels of behavioral isolation among allopatric taxa. Reinforcement between *Ceasia* and *E. caeruleum* has likely occurred, and may have resulted in heightened levels of behavioral isolation among lineages of *Ceasia* that are allopatric to one another but are sympatric with *E. caeruleum*.

Associate Editor: A. Qvarnstrom

Handling Editor: P. Tiffin

## Supporting information


**Figure S1**. Range map for study species. Gray = *Etheostoma caeruleum*, blue = *E. spectabile*, green = *E. fragi*, orange = *E. uniporum*, and purple = *E. burri*.
**Figure S2**. Focal male behavior towards rival males. (a,b) Species set 1F with *E. fragi* as the focal pair and conspecific *Ceasia* rival male, and *E. uniporum* as the allopatric *Ceasia* rival male.
**Figure S3**. Focal male behavior towards rival males. (a,b) Species set 2F with *E. fragi* as the focal pair and conspecific *Ceasia* rival male, and *E. burri* as the allopatric *Ceasia* rival male.
**Figure S4**. Focal male behavior towards rival males. (a,b) Species set 3F with *E. fragi* as the focal pair and conspecific *Ceasia* rival male, and *E. spectabile* as the allopatric *Ceasia* rival male.
**Figure S5**. Rival male behavior towards focal males and focal females. **(**a‐c) Species set 2F with *E. fragi* as the focal pair and conspecific *Ceasia* rival male, and *E. burri* as the allopatric *Ceasia* rival male.
**Figure S6**. Rival male behavior towards focal males and focal females. (a‐c) Species set 3F with *E. fragi* as the focal pair and conspecific *Ceasia* rival male, and *E. spectabile* as the allopatric *Ceasia* rival male.
**Figure S7**. Focal female behavior towards rival males. (a‐b) Species set 1F with *E. fragi* as the focal pair and conspecific *Ceasia* rival male, and *E. uniporum* as the allopatric *Ceasia* rival male.
**Figure S8**. Focal female behavior towards rival males. (a‐b) Species set 2F with *E. fragi* as the focal pair and conspecific *Ceasia* rival male, and *E. burri* as the allopatric *Ceasia* rival male.
**Figure S9**. Focal female behavior towards rival males.
**Figure S10**. STRUCTURE bar plot showing the probability for each individual of belonging to a cluster (See Tables S5‐S8).

**Table S1**. Collection site location information for species used in behavioral assays.
**Table S2**. Number of trials included for each behavior analyzed.
**Table S3**. Collection site location information for species used in genetic analyses.
**Table S4**. Information on number of reads discarded and retained by *process_radtags* in Stacks.
**Table S5**. Results from ANCOVA analyses examining focal female behavior towards rival males.
**Table S6**. Results of the STRUCTURE analysis for the four species of *Ceasia* and *E. caeruleum*.
**Table S7**. Proportion of membership of each pre‐assigned population in each of the two clusters in STRUCTURE for analysis including all four *Ceasia* species and *Etheostoma caeruleum*.
**Table S8**. Results of the STRUCTURE analysis for only the four species of *Ceasia*, excluding *E. caeruleum*.
**Table S9**. Proportion of membership of each pre‐assigned population in each of the two clusters in STRUCTURE for the analysis including all four *Ceasia* species but excluding *Etheostoma caeruleum*.
**Table S10**. K‐means clustering analysis results for variant SNP data set including all five species. Values for pseudo‐*F* statistic calculated in GenoDive (Meirmans and Tienderen 2004).
