# Supplementary material for: Male and female contributions to behavioral isolation in darters as a function of genetic distance and color distance
Source: Evolution. 2017 Sep 14;71(10):2428–44. doi: 10.1111/evo.13321 (PMC5656840; doi:10.1111/evo.13321)
Supplement: Supplementary file 1 — Figure S1. Range map for study species. Gray = Etheostoma caeruleum, blue = E. spectabile, green = E. fragi, orange = E. uniporum, and purple = E. burri. Figure S2. Focal male behavior towards rival males. (a,b) Species set 1F with E. fragi as the focal pair and conspecific Ceasia rival male, and E. uniporum as the allopatric Ceasia rival male. Figure S3. Focal male behavior towards rival males. (a,b) Species set 2F with E. fragi as the focal pair and conspecific Ceasia rival male, and E. burri as the allopatric Ceasia rival male. Figure S4. Focal male behavior towards rival males. (a,b) Species set 3F with E. fragi as the focal pair and conspecific Ceasia rival male, and E. spectabile as the allopatric Ceasia rival male. Figure S5. Rival male behavior towards focal males and focal females. (a‐c) Species set 2F with E. fragi as the focal pair and conspecific Ceasia rival male, and E. burri as the allopatric Ceasia rival male. Figure S6. Rival male behavior towards focal males and focal females. (a‐c) Species set 3F with E. fragi as the focal pair and conspecific Ceasia rival male, and E. spectabile as the allopatric Ceasia rival male. Figure S7. Focal female behavior towards rival males. (a‐b) Species set 1F with E. fragi as the focal pair and conspecific Ceasia rival male, and E. uniporum as the allopatric Ceasia rival male. Figure S8. Focal female behavior towards rival males. (a‐b) Species set 2F with E. fragi as the focal pair and conspecific Ceasia rival male, and E. burri as the allopatric Ceasia rival male. Figure S9. Focal female behavior towards rival males. Figure S10. STRUCTURE bar plot showing the probability for each individual of belonging to a cluster (See Tables S5‐S8). [file EVO-71-2428-s001.docx]

**
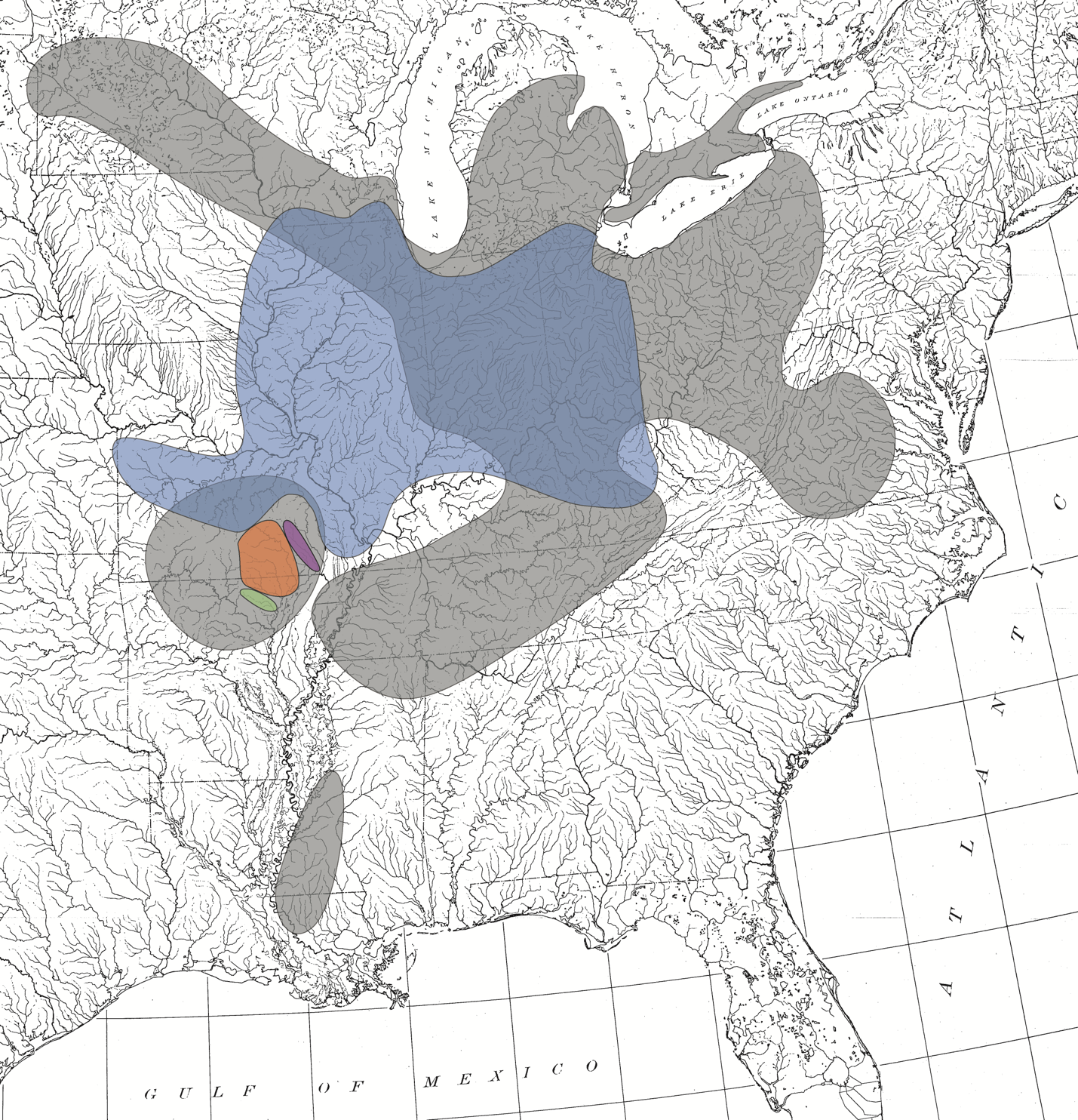
**

**Figure S1.** Range map for study species. Gray = *Etheostoma caeruleum*, blue = *E. spectabile*, green = *E. fragi*, orange = *E. uniporum*, and purple = *E. burri.*

**
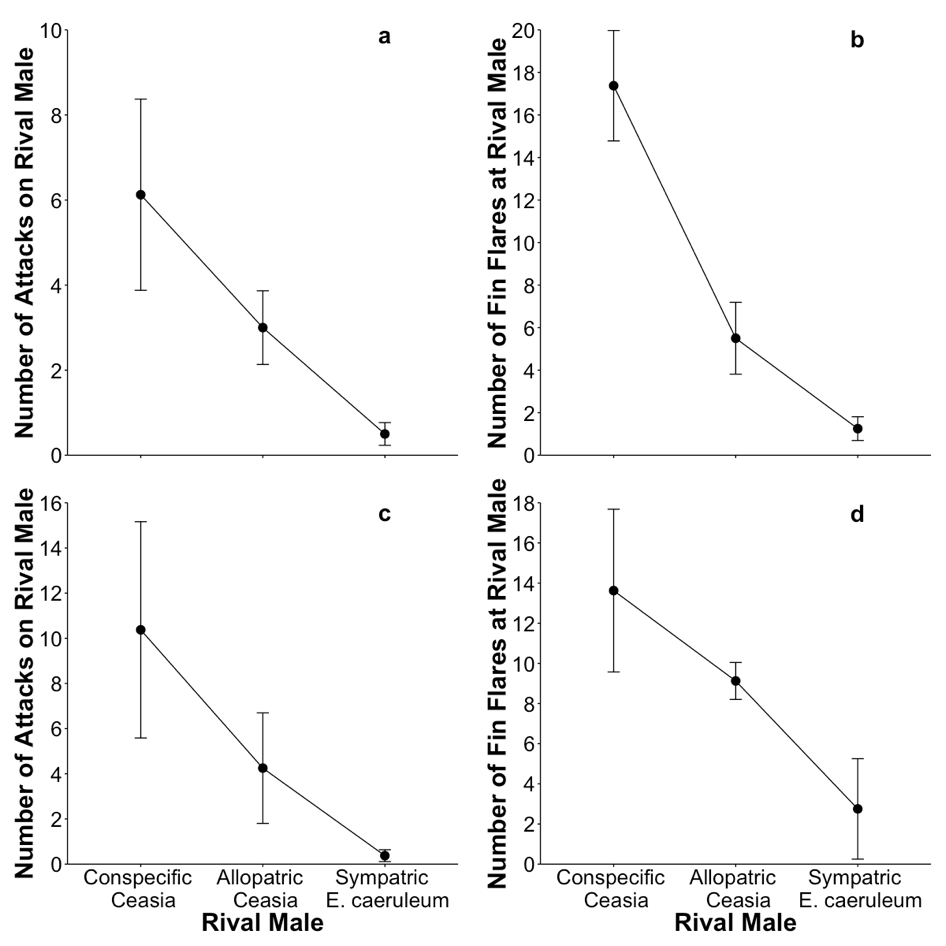
**

**Figure S2.** Focal male behavior towards rival males. (a,b) Species set 1F with *E. fragi* as the focal pair and conspecific *Ceasia* rival male, and *E. uniporum* as the allopatric *Ceasia* rival male*.* (c,d) Species set 1R with *E. uniporum* as the focal pair and conspecific *Ceasia* rival male, and *E. fragi* as the allopatric *Ceasia* rival male*.* (a,c) Focal male attacks on rival male across rival male trial types. (b,d) Focal male fin flares at rival male across rival male trial types.

**
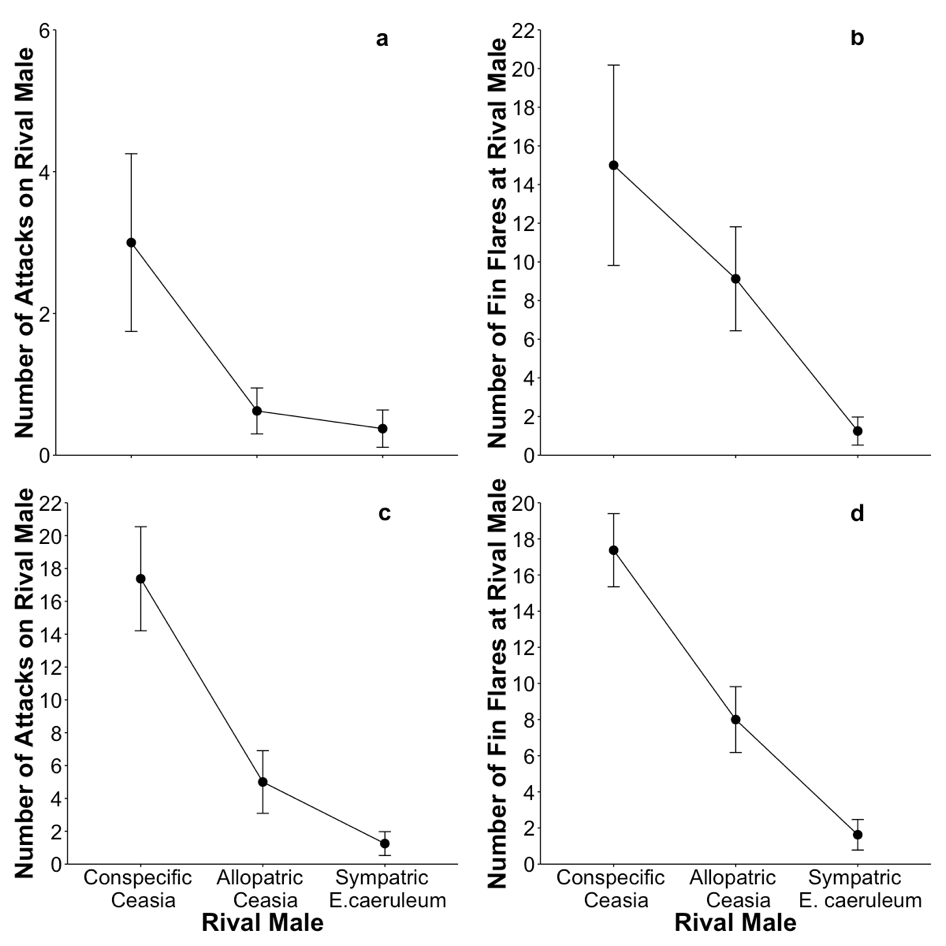
**

**Figure S3.** Focal male behavior towards rival males. (a,b) Species set 2F with *E. fragi* as the focal pair and conspecific *Ceasia* rival male, and *E. burri* as the allopatric *Ceasia* rival male*.* (c,d) Species set 2R with *E. burri* as the focal pair and conspecific *Ceasia* rival male, and *E. fragi* as the allopatric *Ceasia* rival male*.* (a,c) Focal male attacks on rival male across rival male trial types. (b,d) Focal male fin flares at rival male across rival male trial types.

**
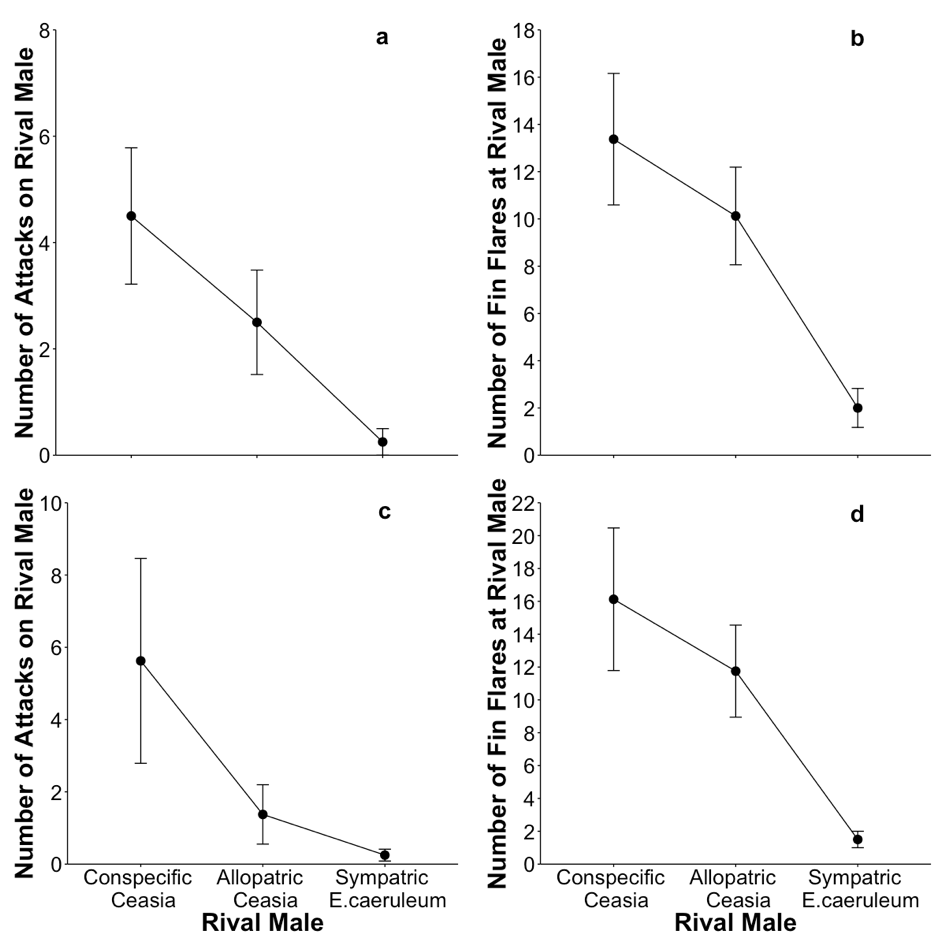
**

**Figure S4.** Focal male behavior towards rival males. (a,b) Species set 3F with *E. fragi* as the focal pair and conspecific *Ceasia* rival male, and *E. spectabile* as the allopatric *Ceasia* rival male*.* (c,d) Species set 3R with *E. spectabile* as the focal pair and conspecific *Ceasia* rival male, and *E. fragi* as the allopatric *Ceasia* rival male*.* (a,c) Focal male attacks on rival male across rival male trial types. (b,d) Focal male fin flares at rival male across rival male trial types.

**
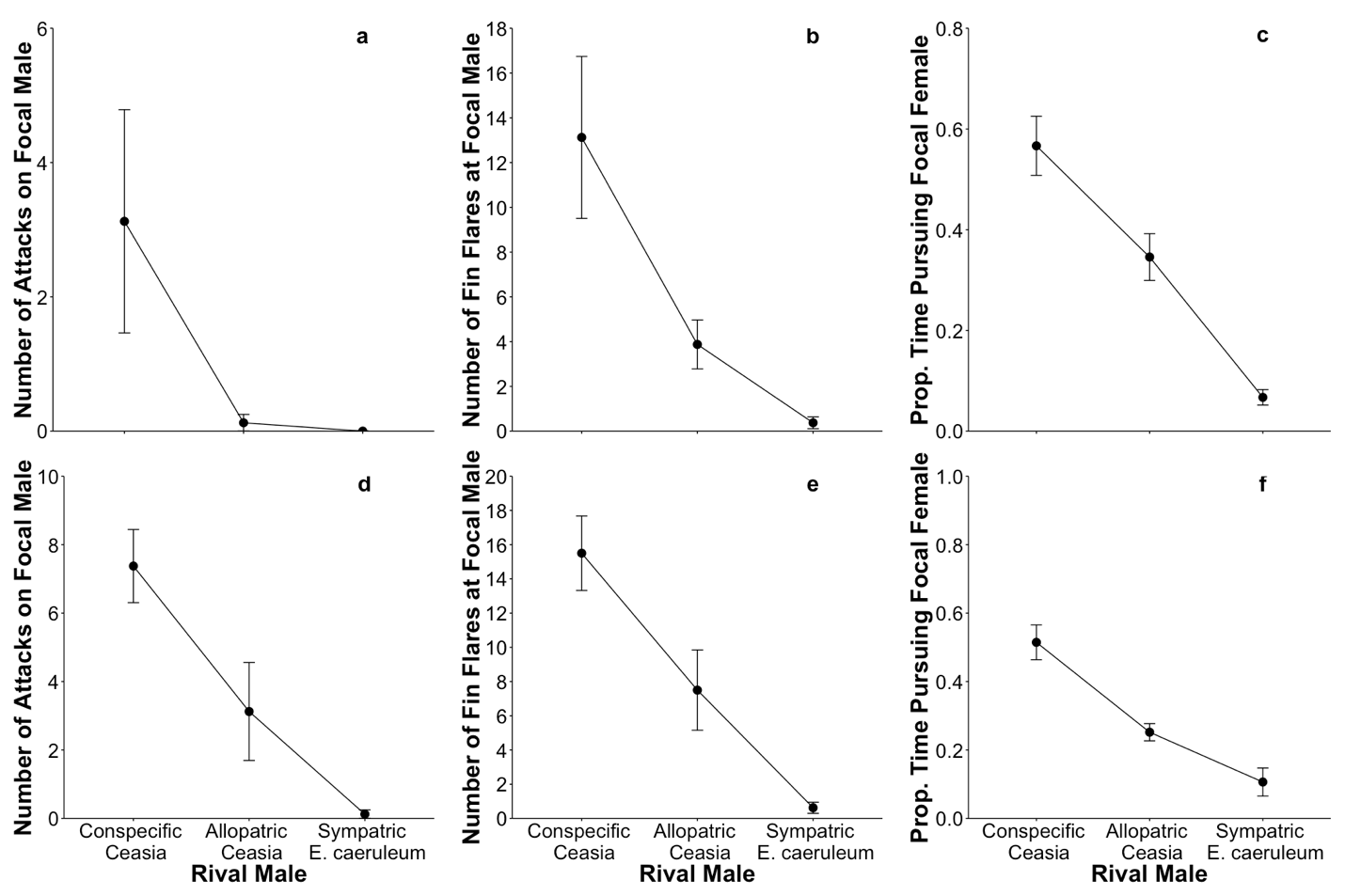
**

**Figure S5.** Rival male behavior towards focal males and focal females. **(**a-c) Species set 2F with *E. fragi* as the focal pair and conspecific *Ceasia* rival male, and *E. burri* as the allopatric *Ceasia* rival male*.* (d-f) Species set 2R with *E. burri* as the focal pair and conspecific *Ceasia* rival male, and *E. fragi* as the allopatric *Ceasia* rival male*.* (a,d) Rival male attacks on focal male. (b,e) Rival male fin flares at focal male. (c,f) Rival male pursuit of focal female.

**
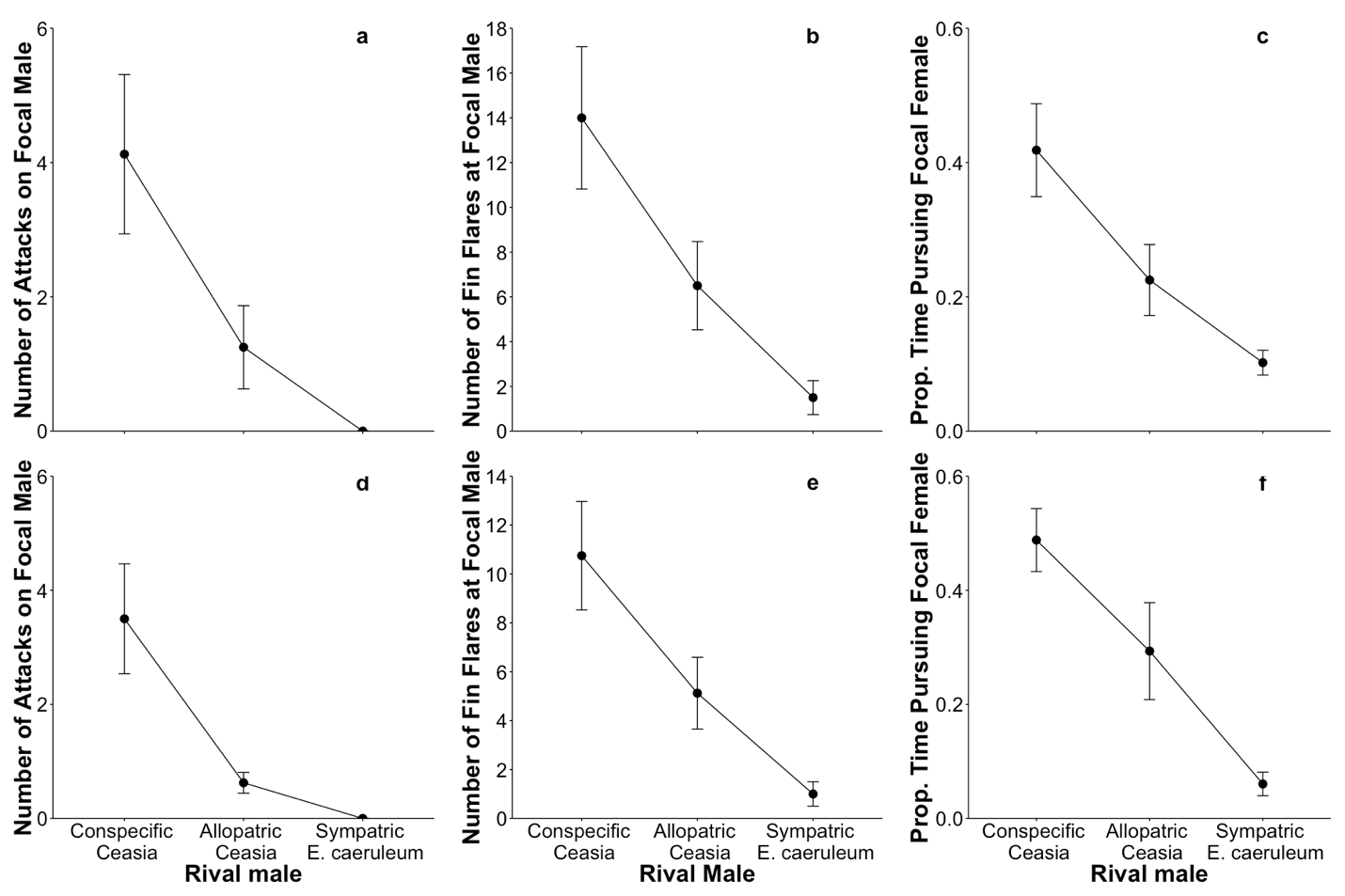
**

**Figure S6.** Rival male behavior towards focal males and focal females. (a-c) Species set 3F with *E. fragi* as the focal pair and conspecific *Ceasia* rival male, and *E. spectabile* as the allopatric *Ceasia* rival male*.* (d-f) Species set 3R with *E. spectabile* as the focal pair and conspecific *Ceasia* rival male, and *E. fragi* as the allopatric *Ceasia* rival male*.* (a,d) Rival male attacks on focal male. (b,e) Rival male fin flares at focal male. (c,f) Rival male pursuit of focal female.

**
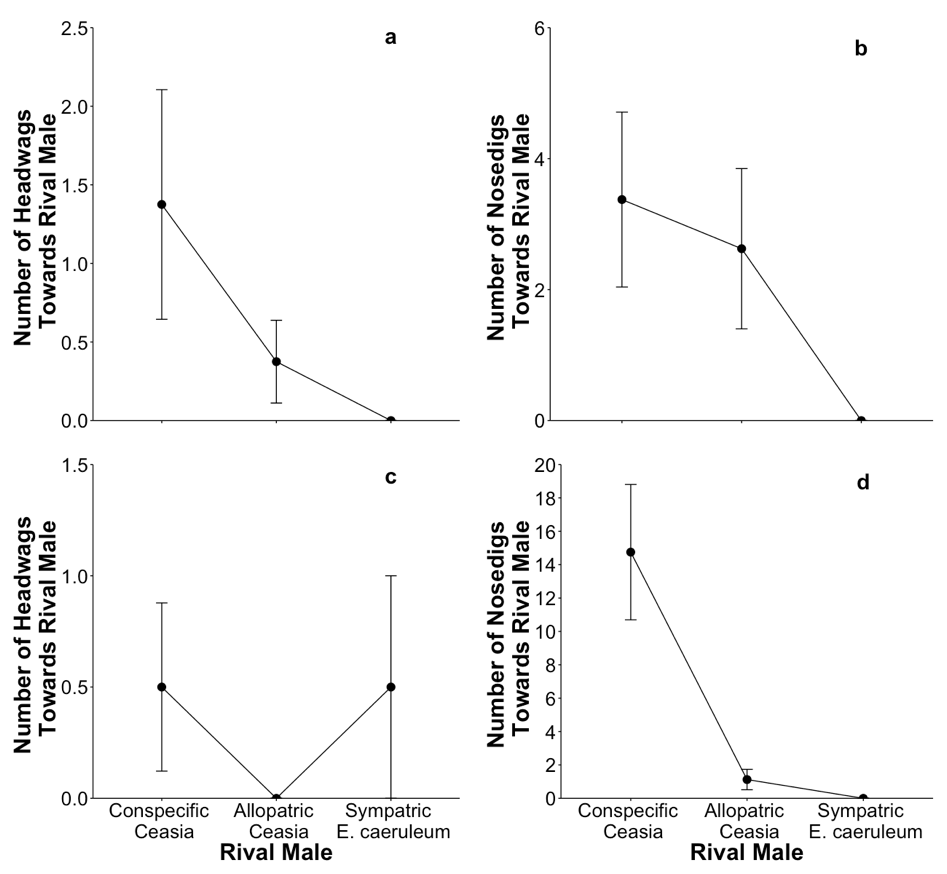
**

**Figure S7.** Focal female behavior towards rival males. (a-b) Species set 1F with *E. fragi* as the focal pair and conspecific *Ceasia* rival male, and *E. uniporum* as the allopatric *Ceasia* rival male*.* (c-d) Species set 1R with *E. uniporum* as the focal pair and conspecific *Ceasia* rival male, and *E. fragi* as the allopatric *Ceasia* rival male*.* (a,c) Focal female headwags towards rival male across rival male trial types. (b,d) Focal female nosedigs towards rival male across rival male trial types.

**
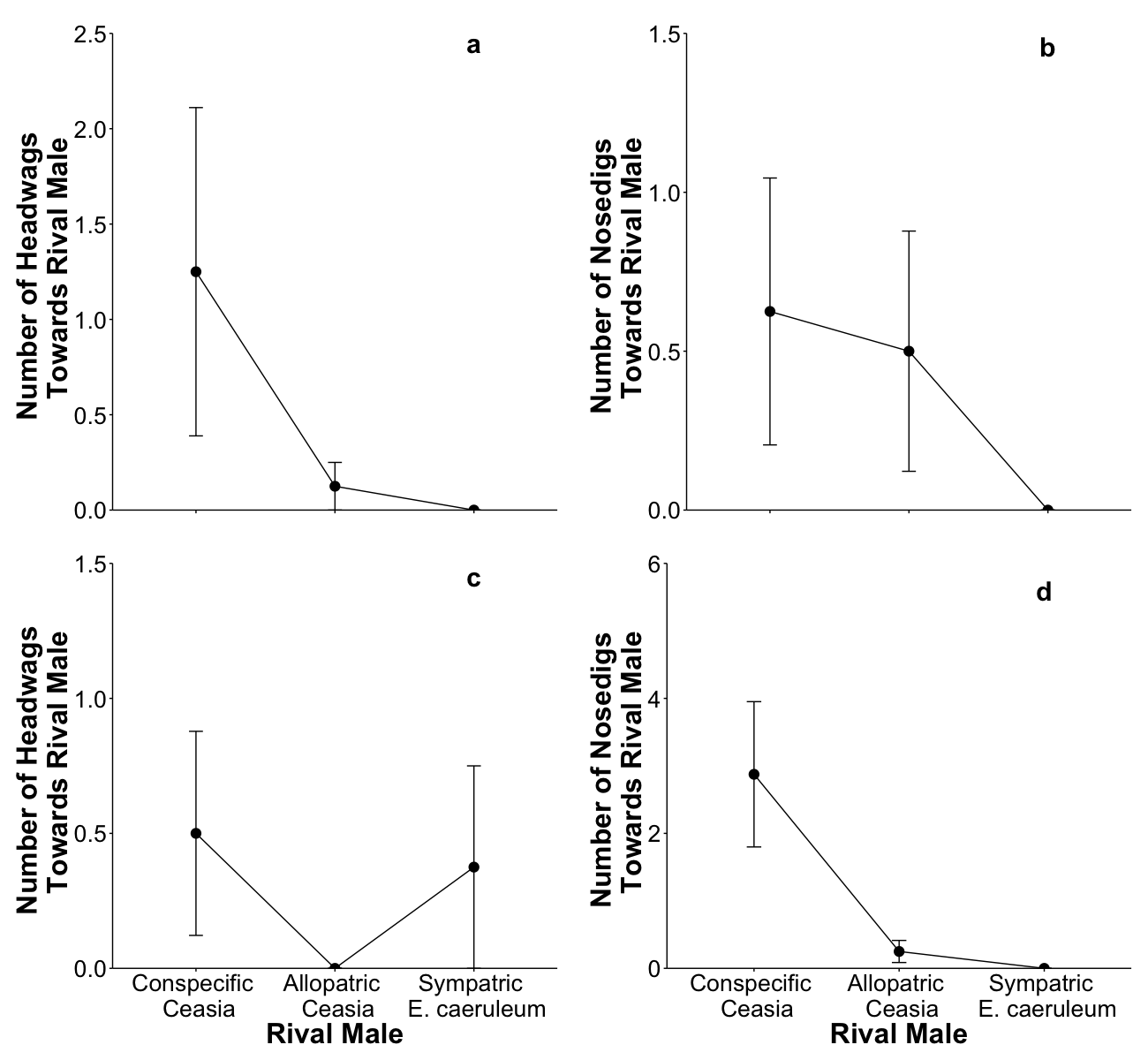
**

**Figure S8.** Focal female behavior towards rival males. (a-b) Species set 2F with *E. fragi* as the focal pair and conspecific *Ceasia* rival male, and *E. burri* as the allopatric *Ceasia* rival male*.* (c-d) Species set 2R with *E. burri* as the focal pair and conspecific *Ceasia* rival male, and *E. fragi* as the allopatric *Ceasia* rival male*.* (a,c) Focal female headwags towards rival male across rival male trial types. (b,d) Focal female nosedigs towards rival male across rival male trial types.

**
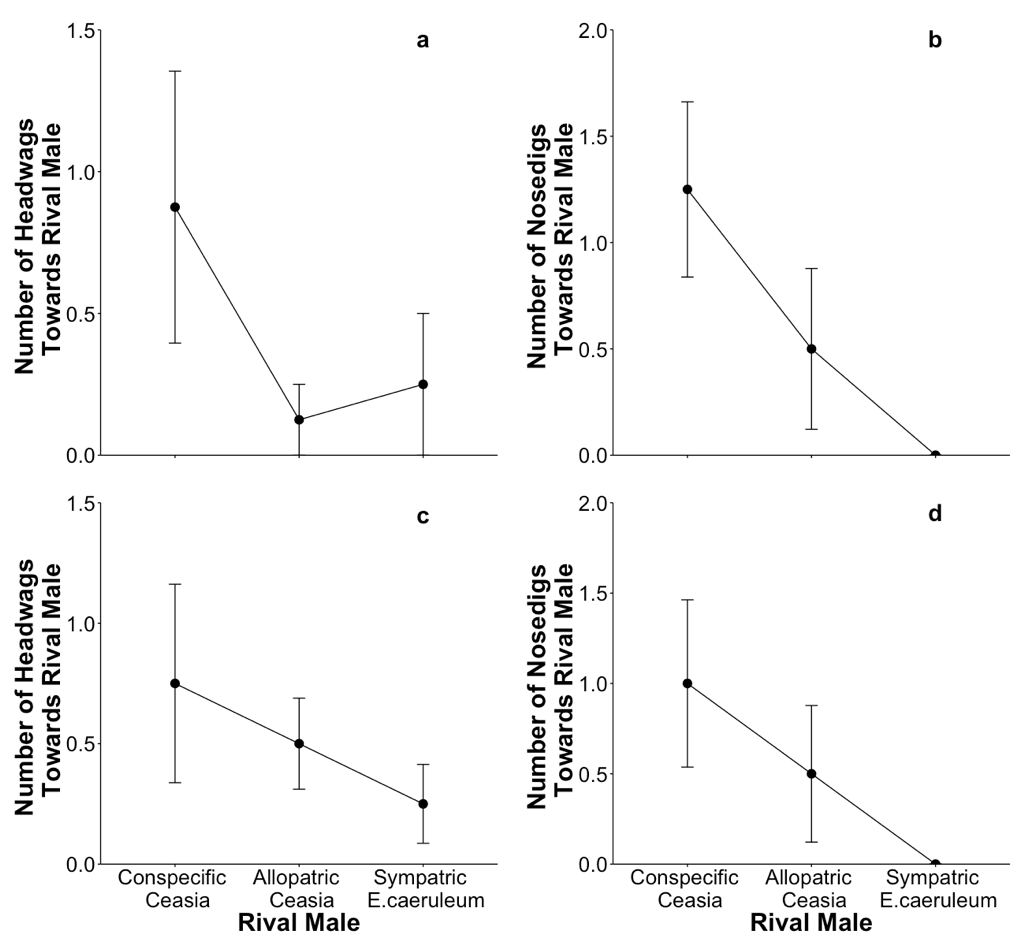
**

**Figure S9.** Focal female behavior towards rival males. (a-b) Species set 3F with *E. fragi* as the focal pair and conspecific *Ceasia* rival male, and *E. spectabile* as the allopatric *Ceasia* rival male*.* (c-d) Species set 3R with *E. spectabile* as the focal pair and conspecific *Ceasia* rival male, and *E. fragi* as the allopatric *Ceasia* rival male*.* (a,c) Focal female headwags towards rival male across rival male trial types. (b,d) Focal female nosedigs towards rival male across rival male trial types.


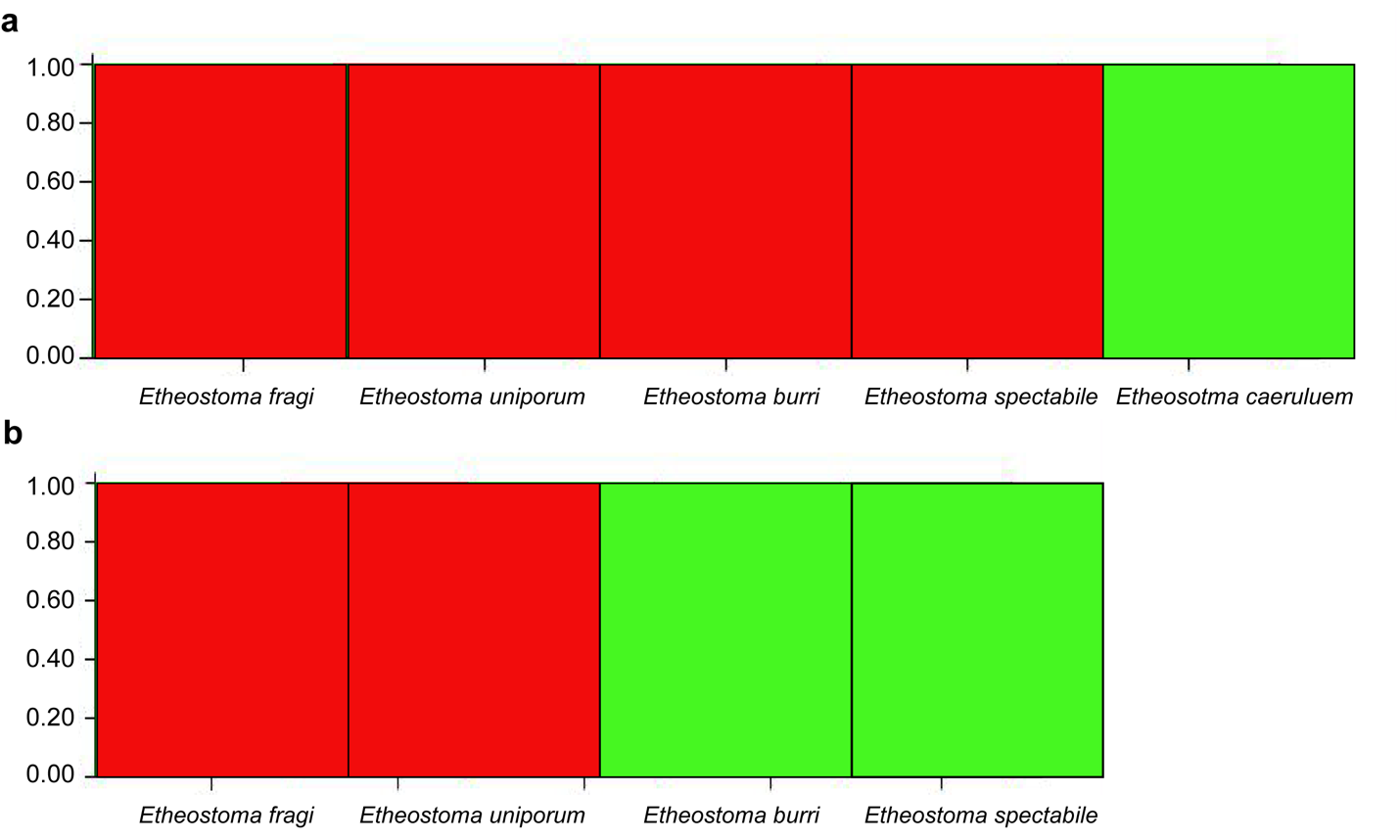


**Figure S10.** STRUCTURE bar plot showing the probability for each individual of belonging to a cluster (See Tables S5-S8). (a) STRUCTURE analysis including all four *Ceasia* species and the more distantly related *Etheostoma caeruleum*. The optimal number of distinct clusters (K) determined to be two using the Delta K method (Evano et al. 2005). (b) STRUCTURE analysis only including the four *Ceasia* species and excluding *E. caeruleum.* The optimal number of distinct clusters (K) determined to be two using the Delta K method (Evano et al. 2005).
