# Supplementary material for: Male and female contributions to behavioral isolation in darters as a function of genetic distance and color distance
Source: Evolution. 2017 Sep 14;71(10):2428–44. doi: 10.1111/evo.13321 (PMC5656840; doi:10.1111/evo.13321)
Supplement: Supplementary file 2 — Table S1. Collection site location information for species used in behavioral assays. Table S2. Number of trials included for each behavior analyzed. Table S3. Collection site location information for species used in genetic analyses. Table S4. Information on number of reads discarded and retained by process_radtags in Stacks. Table S5. Results from ANCOVA analyses examining focal female behavior towards rival males. Table S6. Results of the STRUCTURE analysis for the four species of Ceasia and E. caeruleum. Table S7. Proportion of membership of each pre‐assigned population in each of the two clusters in STRUCTURE for analysis including all four Ceasia species and Etheostoma caeruleum. Table S8. Results of the STRUCTURE analysis for only the four species of Ceasia, excluding E. caeruleum. Table S9. Proportion of membership of each pre‐assigned population in each of the two clusters in STRUCTURE for the analysis including all four Ceasia species but excluding Etheostoma caeruleum. Table S10. K‐means clustering analysis results for variant SNP data set including all five species. Values for pseudo‐F statistic calculated in GenoDive (Meirmans and Tienderen 2004). [file EVO-71-2428-s002.docx]

**Table S1.** Collection site location information for species used in behavioral assays.

| **Species** | **Latitude, Longitude** | **Collection Site Drainage Information** |
| --- | --- | --- |
| *E. fragi* (strawberry darter) | 36.304214, -91.927684 | Rose Branch tributary of Strawberry River, White River Drainage, Salem, AR |
| *E. uniporum* (current darter) | 36.250560, -91.359318 | Unnamed tributary of Spring River, White River Drainage, Williford, AR |
| *E. burri* (brook darter) | 37.146415, -90.907459 | North Fork Webb Creek, Black River Drainage, Logan Township, MO |
| *E. spectabile* (orangethroat darter) | 40.089035, -88.143440 | Unnamed tributary of Salt Fork Vermilion River, Wabash River Drainage, Champaign, IL |
| *E. caeruleum* (Mississippi River Corridor clade rainbow darter)* | 36.065396, -91.610420 | Mill Creek tributary of Strawberry River, White River Drainage, Evening Shade, AR |
| *E. caeruleum* (Eastern clade rainbow darter)** | 40.055556, -88.091667 | Unnamed tributary of Salt Fork Vermilion River, Wabash River Drainage, Champaign, IL |

*Used as sympatric rival male in trials where *E. fragi*, *E. uniporum*, and *E. burri* served as focal pair.

** Used as sympatric rival male in trials where *E. spectabile* served as focal pair.

**Table S2.** Number of trials included for each behavior analyzed.

| **Trial Set** | **Nosedigs** | **Headwags** | **Male pursuit** | **Attacks** | **Fin flares** |
| --- | --- | --- | --- | --- | --- |
| 1F and 1R | 19 | 38 | 48 | 48 | 48 |
| 2F and 2R | 17 | 30 | 48 | 48 | 48 |
| 3F and 3R | 16 | 29 | 48 | 48 | 48 |

**Table S3.** Collection site location information for species used in genetic analyses.

| **Species** | **Latitude, Longitude** | **Collection Site Drainage Information** |
| --- | --- | --- |
| *E. fragi* (strawberry darter) | 36.304214, -91.927684 | Rose Branch tributary of Strawberry River, White River Drainage, Salem, AR |
| *E. uniporum* (current darter) | 37.057146, -91.022982 | Pine Valley Creek, Current River, White River Drainage, Van Buren, MO |
| *E. burri* (brook darter) | 37.146415, -90.907459 | North Fork Webb Creek, Black River Drainage, Logan Township, MO |
| *E. spectabile* (orangethroat darter) | 40.089035, -88.143440 | Unnamed tributary of Salt Fork Vermilion River, Wabash River Drainage, Champaign, IL |
| *E. caeruleum* (Mississippi River Corridor clade rainbow darter) | 37.031917, -91.036867 | Pine Valley Creek, Current River, White River Drainage, Van Buren, MO |

**Table S4.** Information on number of reads discarded and retained by *process_radtags* in Stacks.

| **Total Number of Reads** | **Read Length (bp)** | **Reason Read was Discarded** | | | **Reads**  **Retained** | **Percent of Reads Retained** |
| --- | --- | --- | --- | --- | --- | --- |
|  |  | **Ambiguous Barcodes** | **Low Quality** | **Ambiguous RAD-Tag** |  |  |
| 251,420,894 | 100 | 15,257,833 | 6,199,861 | 9,410,201 | 220,552,999 | 87.72 |

**Table S5.** Results from ANCOVA analyses examining focal female behavior towards rival males. The table headings (A-C) list the two *Ceasia* species in the species set (*E. fragi* and a heterospecific allopatric *Ceasia* species) followed by the sympatric, distantly related *E. caeruleum.*

| **A. *E. fragi* – *E. uniporum – E. caeruleum* (1F and 1R)** | | | |
| --- | --- | --- | --- |
| Variable: Headwags towards rival male | **df** | **Test statistic** | **p** |
| Rival male identity | 2,31 | 2.9876 | 0.0651 |
| Focal pair identity | 1,31 | 1.4044 | 0.2450 |
| Pursuit by rival male | 1,31 | 47.235 | **<0.0001** |
| Rival male identity * focal pair identity | 2,31 | 1.5383 | 0.2307 |
| Variable: Nosedigs towards rival male | **df** | **Test statistic** | **p** |
| Rival male identity | 2,12 | 0.7963 | 0.4735 |
| Focal pair identity | 1,12 | 0.4070 | 0.5355 |
| Pursuit by rival male | 1,12 | 23.753 | **<0.001** |
| Rival male identity * focal pair identity | 2,12 | 0.0160 | 0.9841 |
|  |  |  |  |
| **B. *E. fragi - E. burri - E. caeruleum* (2F and 2R)** | | | |
| Variable: Headwags towards rival male | **df** | **Test statistic** | **p** |
| Rival male identity | 2,23 | 0.1731 | 0.8421 |
| Focal pair identity | 1,23 | 2.0644 | 0.1642 |
| Pursuit by rival male | 1,23 | 3.7075 | 0.0666 |
| Rival male identity * focal pair identity | 2,23 | 0.4727 | 0.6292 |
| Variable: Nosedigs towards rival male | **df** | **Test statistic** | **p** |
| Rival male identity | 2,9 | 0.3383 | 0.7217 |
| Focal pair identity | 1,9 | 4.002 | 0.0765 |
| Pursuit by rival male | 1,9 | 0.1709 | 0.6890 |
| Rival male identity * focal pair identity | 2,9 | 1.5930 | 0.2557 |
| **C. *E. fragi* – *E. spectabile – E. caeruleum* (3F and 3R)** | | | |
| Variable: Headwags towards rival male | **df** | **Test statistic** | **p** |
| Rival male identity | 2,22 | 1.1741 | 0.3277 |
| Focal pair identity | 1,22 | 3.6363 | 0.0697 |
| Pursuit by rival male | 1,22 | 16.407 | **<0.001** |
| Rival male identity * focal pair identity | 2,22 | 0.4653 | 0.6340 |
| Variable: Nosedigs towards rival male | **df** | **Test statistic** | **p** |
| Rival male identity | 2,9 | 1.4891 | 0.2763 |
| Focal pair identity | 1,9 | 1.0152 | 0.3400 |
| Pursuit by rival male | 1,9 | 9.2894 | **0.0138** |
| Rival male identity * focal pair identity | 2,9 | 0.4668 | 0.6414 |

**Table S6.** Results of the STRUCTURE analysis for the four species of *Ceasia* and *E. caeruleum*. Using the Delta K method for estimating K (Evanno et al. 2005), the optimal value of K is 2. Calculations were performed using Structure Harvester (Earl and vonHoldt 2012).

| **K** | **Reps** | **Mean LnP(K)** | **SD LnP(K)** | **Ln'(K)** | **\|Ln''(K)\|** | **Delta K** |
| --- | --- | --- | --- | --- | --- | --- |
| 1 | 50 | -38618.65 | 3.69 | - | - | - |
| 2 | 50 | -27344.16 | 907.97 | 11274.49 | 14299.14 | **15.75** |
| 3 | 50 | -30368.81 | 67588.97 | -3024.65 | 22201.82 | 0.33 |
| 4 | 50 | -11191.64 | 2045.29 | 19177.17 | 17988.48 | 8.80 |
| 5 | 50 | -10002.95 | 2559.90 | 1188.69 | 798.27 | 0.31 |
| 6 | 50 | -9612.53 | 2259.66 | 390.42 | 620.44 | 0.27 |
| 7 | 50 | -8601.66 | 2099.72 | 1010.86 | 870.17 | 0.41 |
| 8 | 50 | -8460.97 | 2036.82 | 140.69 | - | - |

**Table S7.** Proportion of membership of each pre-assigned population in each of the two clusters in STRUCTURE for analysis including all four *Ceasia* species and *Etheostoma caeruleum*.

| **Species** | **Cluster 1** | **Cluster 2** | **Number of Individuals** |
| --- | --- | --- | --- |
| *Etheostoma burri* | 1.00 | 0.00 | 12 |
| *Etheostoma spectabile* | 1.00 | 0.00 | 12 |
| *Etheostoma fragi* | 1.00 | 0.00 | 12 |
| *Etheostoma uniporum* | 1.00 | 0.00 | 11 |
| *Etheostoma caeruleum* | 0.00 | 1.00 | 12 |

**Table S8.** Results of the STRUCTURE analysis for only the four species of *Ceasia,* excluding *E. caeruleum*. Using the Delta K method for estimating K (Evanno et al. 2005), the optimal value of K is 2. Calculations were performed using Structure Harvester (Earl and vonHoldt 2012).

| **K** | **Reps** | **Mean LnP(K)** | **SD LnP(K)** | **Ln'(K)** | **\|Ln''(K)\|** | **Delta K** |
| --- | --- | --- | --- | --- | --- | --- |
| 1 | 50 | -38562.30 | 3.31 | - | - | - |
| 2 | 50 | -22179.45 | 2537.34 | 16382.85 | 142651.47 | **56.22** |
| 3 | 50 | -148448.07 | 959138.00 | -126268.62 | 334482.29 | 0.35 |
| 4 | 50 | -609198.99 | 2396921.89 | -460750.91 | 902296.72 | 0.38 |
| 5 | 50 | -167653.18 | 901024.69 | 441545.80 | 611030.98 | 0.68 |
| 6 | 50 | -337138.36 | 1647994.64 | -169485.18 | 298040.65 | 0.18 |
| 7 | 50 | -208582.89 | 1418176.47 | 128555.47 | - | - |

**Table S9.** Proportion of membership of each pre-assigned population in each of the two clusters in STRUCTURE for the analysis including all four *Ceasia* species but excluding *Etheostoma caeruleum.*

| **Species** | **Cluster 1** | **Cluster 2** | **Number of Individuals** |
| --- | --- | --- | --- |
| *Etheostoma burri* | 0.00 | 1.00 | 12 |
| *Etheostoma spectabile* | 0.00 | 1.00 | 12 |
| *Etheostoma fragi* | 1.00 | 0.00 | 12 |
| *Etheostoma uniporum* | 1.00 | 0.00 | 11 |

**Table S10.** K-means clustering analysis results for variant SNP data set including all five species. Values for pseudo-*F* statistic calculated in GenoDive (Meirmans and Tienderen 2004).

| K | Pseudo-*F* |
| --- | --- |
| 1 | 0.000 |
| 2 | 23.95 |
| 3 | 36.07 |
| 4 | 47.73 |
| 5 | **59.88** |
| 6 | 51.44 |
| 7 | 46.13 |
| 8 | 41.06 |
